# Association Between Socio-Demographic, Behavioural, and Health-Related Factors and Fruit, Vegetable, and Salt Consumption Among Adults Aged 18–69 Years in Kazakhstan: A Cross-Sectional Study

**DOI:** 10.3390/nu18071154

**Published:** 2026-04-03

**Authors:** Marat Shoranov, Anel Ibrayeva, Mirzakarim Alchinbayev, Bolat Sadykov, Yerlan Ismoldayev, Asset Izdenov, Ildar Fakhradiyev, Sergey Lee, Shynar Tanabayeva

**Affiliations:** 1 Department of Medicine, S.D. Asfendiyarov Kazakh National Medical University, Almaty 050000, Kazakhstan; 2 Ministry of Healthcare of the Republic of Kazakhstan, Astana 010000, Kazakhstan; 3 College of Medicine, Korea University, Seoul 02841, Republic of Korea

**Keywords:** nutrition, fruit and vegetable consumption, salt consumption practices, Kazakhstan, STEPS survey

## Abstract

**Background/Objectives**: Low fruit and vegetable consumption and unhealthy salt-related behaviours are important modifiable risk factors for non-communicable diseases. Nationally representative data on these dietary patterns in Kazakhstan remain limited. We aimed to assess fruit, vegetable, and salt-related behaviours among adults aged 18–69 years and examine the socio-demographic and geographic factors associated with inadequate fruit and vegetable consumption and unfavourable salt-related knowledge, attitudes, and behaviours. **Methods**: We conducted a nationally representative cross-sectional survey of 6720 adults across all 17 administrative units of Kazakhstan in 2021–2022 using multistage stratified cluster sampling and the WHO STEPS questionnaire. Fruit and vegetable consumption, as well as salt-related knowledge, attitudes, and behaviours, were assessed by interview. Associations were examined using weighted logistic regression. **Results**: Mean fruit and vegetable consumption was 3.0 (SD 2.3) servings/day, and only 15.7% of respondents met the WHO recommendation of ≥5 servings/day. Women were more likely than men to meet the WHO recommendation (17.9% vs. 13.4%), and men had lower adjusted odds of adequate fruit and vegetable consumption (aOR 0.72, 95% CI 0.62–0.84). Adequate fruit and vegetable consumption was most common in southern regions and least common in northern and urban areas. Although 80.6% of participants were aware of the health risks of high salt consumption, only 41.7% considered salt reduction very important, while 64.6% reported adding salt during cooking and 39.6% at the table, and 29.2% frequently consumed salty processed foods. Less favourable salt-related patterns were more common among men, younger adults, less educated respondents, smokers, and residents of several administrative units. **Conclusions**: Adults in Kazakhstan show insufficient fruit and vegetable consumption and suboptimal salt-related behaviours. Targeted, geographically adapted, multisectoral measures are needed to support healthier dietary practices.

## 1. Introduction

Unhealthy diet is among the leading modifiable causes of chronic non-communicable diseases (NCDs) worldwide. According to the Global Burden of Disease estimates, in 2019 dietary risks accounted for about 28.5% of risk-attributable NCD deaths worldwide [1], with key elements of the “dietary” burden being insufficient intake of vegetables and fruits [2] and excessive salt consumption [3]. It has been proven that higher consumption of fruits and vegetables is associated with reduced mortality [4].

The World Health Organisation (WHO) recommends consuming at least five servings (≈400 g) of vegetables and fruits per day [5] and limiting salt intake to less than 5 g per day (less than 2 g of sodium) to reduce the risk of arterial hypertension and cardiovascular diseases [6]. However, in many countries, especially those with low and middle incomes, the actual consumption of vegetables and fruits is significantly below recommended levels [7], while salt intake remains consistently high [8]. Overall, 77.6% of men and 78.4% of women, mainly from low- and middle-income countries, consume fewer than the minimally recommended five servings of fruits and vegetables per day [9].

In Central Asia and Eastern/Central Europe, some of the highest mortality rates associated with excessive sodium intake have been recorded. In 2010, sodium-attributable cardiovascular mortality in these regions was 2536 deaths per 1,000,000 adults per year, accounting for 12.9% of all cardiovascular deaths [10].

According to studies, about 92% of adults in Kazakhstan do not meet the recommended daily level of fruit and vegetable consumption [11], while the average daily salt intake in the country is ~17 g, which is almost four times higher than the acceptable norm and is one of the highest levels in the world [12]. In Kazakhstan, dietary behaviour is shaped not only by food availability and affordability, but also by regional and cultural food traditions, including the common use of salt in home cooking, preserved foods, and processed meat-based dishes.

Despite the seriousness of the problem, the available studies are limited in geography and sample size [13]. A study conducted by the Kazakh Academy of Nutrition on salt intake in Kazakhstan covered only two cities (Almaty and Kyzylorda) and included 472 participants [14]. Despite its undeniable importance for understanding the level of salt consumption among the population, the limited coverage and small sample size do not allow its data to be considered representative for the entire country. Kazakhstan still belongs to the group of WHO European Region countries for which national nutrition data are lacking [15]. Most studies have focused on certain subgroups of the population [16,17,18,19] and macroeconomic indicators [20], while nationally representative evidence on adult fruit, vegetable, and salt-related behaviours in Kazakhstan remains limited, particularly with respect to socio-demographic and regional variation.

Despite recognizing excessive salt consumption as a serious health threat, the implementation of salt reduction policies in Kazakhstan remains insufficient. There is no regular monitoring of salt intake and sodium content in food in the country, no measures have been taken to reformulate products to reduce salt content, mandatory sodium labeling and front-of-pack labeling systems have not been introduced. There are also no restrictions on advertising unhealthy foods for children, while awareness campaigns and school programs are only partially implemented. A similar situation is observed with fruit and vegetable consumption: daily consumption remains below the recommended level, while insufficient efforts are made to stimulate demand, ensure affordability, and promote sustainable healthy eating habits. As a result, the key WHO “best buys”—reformulation, labeling, education, and supportive food environments—require large-scale and systematic implementation [21].

Ineffective management of dietary factors undermines efforts to control NCDs in the country. The probability of premature death (before the age of 70) from the four main NCDs in Kazakhstan had already reached 27% in 2016; more than half of the adult population is overweight or obese, and the financial burden of NCDs is estimated at 4.5% of GDP [15].

Against this background, identifying the socio-demographic predictors of actual dietary behaviour of the population remains an urgent task. We hypothesized that inadequate fruit and vegetable consumption and unfavourable salt-related behaviours would vary across socio-demographic and regional groups. The aim of the present study is to assess fruit, vegetable, and salt consumption among adults aged 18–69 years in Kazakhstan and to identify demographic, socio-economic, and regional factors associated with inadequate fruit and vegetable consumption and unfavourable salt-related outcomes, using data from a nationally representative survey conducted in 2021–2022. The results will help to substantiate priority groups and mechanisms for targeted interventions in the field of healthy nutrition.

## 2. Materials and Methods

### 2.1. Study Design and Sample

We conducted a cross-sectional, nationally representative survey of adults in Kazakhstan from October 2021 to May 2022. A total of 6720 individuals aged 18–69 years were selected using a multistage, stratified cluster design across all 17 administrative units. Survey weights (accounting for unequal probabilities of selection and non-response), with post-stratification by sex, age group, and region, were applied to yield estimates representative of the non-institutionalized adult population of Kazakhstan aged 18–69 years at the national level.

### 2.2. Study Context

Kazakhstan, located in Central Asia, is administratively subdivided into 14 regions, three cities of republican significance (Astana, Almaty, and Shymkent), and 177 districts. The majority of the country’s 20 million inhabitants live in urban areas, despite its low population density of 6 people per square kilometer (https://stat.gov.kz/, accessed on 2 September 2025).

### 2.3. Sampling

In this study, a multistage cluster sampling design was used with stratification by administrative unit and age group. The sample size was calculated using the World Health Organization’s STEPS sample size calculator (Excel-based tool) with the following parameters: a 95% confidence level (Z = 1.96), an assumed prevalence of 0.5, a standard error of 0.05, a design effect of 1.5, and an anticipated response rate of 70%. Based on these assumptions, the minimum required sample size was estimated at n = 6585.

Sampling was conducted in three stages, with clustering at each stage. At the first stage, primary sampling units were defined as districts and urban centers and were selected proportionally across the administrative units of Kazakhstan using data from the Bureau of National Statistics and the Agency for Strategic Planning and Reforms of the Republic of Kazakhstan. At the second stage, Primary Health Care (PHC) facilities were selected as secondary sampling units using the national PHC registry of the Republican Centre for Healthcare Development, with probability proportional to the patient population served by each PHC. At the third stage, households and individual respondents were selected. The target number of households per PHC was estimated as 6585/240 ≈ 28.

To account for possible non-response and the use of reserve households, the final planned sample size was increased to 6720 adults. A roster of households linked to each selected PHC was compiled, and households were randomly selected using the Randhold.xls tool. Within each selected household, one eligible respondent was chosen using the Kish method. Eligible participants were adults in the target age range residing in the selected households during the survey period. Individuals were excluded if they were outside the target age range, did not reside in the selected household during the survey period, were unable to complete the interview or examination, or declined participation. Participation was voluntary, and written informed consent was obtained before the interview and examination.

### 2.4. Handling of Missing Data

The initial sample included 6720 respondents. For descriptive statistics, proportions and means were calculated using the total sample size as the denominator, while the valid number of observations was reported for each variable. In regression models, participants were excluded only if data on the corresponding dependent variable were missing, so the analytical sample size varied across outcomes.

For categorical independent variables with missing values, we used the missing-indicator method: a separate “no data” category was added to retain individuals with incomplete covariate information in the analysis. For continuous predictors with a small proportion of missing values (<5%), regression analyses were performed using complete cases for the corresponding variable.

### 2.5. Data Collection

Before the survey, data collection teams received training on interview techniques and physical/biochemical measurements. Interviewers explained the study’s goals to each household and obtained informed consent. Face-to-face interviews were conducted at PHC facilities, and physical/biochemical measurements were taken on the same day. No financial compensation was provided for participation. Data collection in 2021–2022 was conducted under COVID-19 mitigation (symptom screening, PPE, ventilation/outdoor settings where feasible, appointment-based visits, brief pauses during local surges).

### 2.6. Data Variables

The study followed the standardized WHO STEPwise approach [22]. The questionnaire was administered in Russian or Kazakh, according to participants’ preferences. In both cases, items were adapted from the original English WHO STEPS instrument using a standard forward–backward translation procedure. First, two bilingual physicians independently translated the English questionnaire into Russian and Kazakh, respectively, and any discrepancies between the forward translations were reconciled into single Russian and Kazakh versions. These preliminary versions were then back-translated into English by independent bilingual translators. The back-translations were compared with the original English questionnaire, and iterative revisions were undertaken until the research team agreed that both the Russian and Kazakh versions were conceptually equivalent to the source instrument.

In Step 1, trained interviewers collected socio-demographic data (age, sex, ethnicity, place of residence, education level, marital status, occupation) and information on behavioural risk factors (tobacco use, alcohol consumption).

Step 2 included physical measurements. Anthropometry followed WHO STEPS SOPs. Height was measured with a portable stadiometer to 0.1 cm, weight with a calibrated digital scale to 0.1 kg (light clothing, no shoes), and waist/hip circumferences with a non-stretch tape to 0.1 cm. Each measure was taken twice (third if discrepancy > 0.5 kg or >0.5 cm) and averaged. Blood pressure was measured using an automated oscillometric device A&D UA-888. After ≥5 min of seated rest (no caffeine/smoking/exercise ≥ 30 min), three readings were obtained at 1 min intervals; the mean of the 2nd and 3rd readings (mmHg) was used for analysis. Body mass index (BMI) was calculated and categorized into underweight (<18.5 kg/m^2^), normal weight (18.5–24.9 kg/m^2^), overweight (25–29.9 kg/m^2^), and obesity (≥30 kg/m^2^) [23]. The hypertension status was classified as follows: normotension: mean systolic blood pressure (SBP) < 120 mmHg and diastolic blood pressure (DBP) < 80 mmHg; prehypertension: mean SBP ≥ 120–139 mmHg and DBP ≥ 80–<89 mmHg; hypertension: mean SBP ≥ 140 mmHg and/or DBP ≥ 90 mmHg and/or treatment with antihypertensive drugs within 2 weeks [24].

Although biochemical samples were collected as part of the broader STEPS protocol, biochemical variables were not included in the present analysis and are therefore not described in detail here.

### 2.7. Survey Contents

A standardized questionnaire, the WHO STEPwise approach to surveillance was employed [22]. The survey criteria used were as follows: gender, age, place of residence, ethnicity, educational status, marital status, occupation. A respondent who answered “Yes” to “Do you currently smoke any tobacco products?” was considered a current smoker. Heavy episodic drinking (HED) is defined as “drinking at least 60 g or more of pure alcohol on at least one occasion in the past 30 days” (WHO) [25].

The questionnaire included questions on fruit and vegetable consumption, including frequency (number of days in a typical week) and the number of servings consumed on such a day. To assess servings, visual materials were used: cards with images (for raw products) and measuring cups (for cooked dishes). Adequate fruit and vegetable consumption was defined as a total typical daily consumption of at least 5 servings of fruits and vegetables (WHO).

Within the WHO STEPS questionnaire, questions were used to assess respondents’ knowledge, attitude, and behaviour (KAB) regarding salt consumption. The questionnaire included six questions grouped into three KAB categories (knowledge, attitude, behaviour) in accordance with the WHO STEPS methodology. Knowledge was assessed based on respondents’ conviction that excessive salt consumption may cause serious health problems. Attitude was reflected in the self-assessment of the amount of salt consumed and the subjective importance of limiting it in the diet. Respondents’ behaviour was assessed through three questions: the frequency of adding salt or salty sauces to ready-to-eat food, the use of salt or salty seasonings during home cooking, and the consumption of processed foods with high sodium content.

### 2.8. Outcome Measures

The primary outcomes of interest were defined as follows:

– Adequate fruit and vegetable consumption: Meeting the WHO recommendation of ≥5 servings of fruits and vegetables per day (yes/no).
– Salt-related knowledge: Acknowledging that high salt consumption can cause serious health problems (yes/no).
  Salt-related attitudes toward salt reduction were assessed using two items: (1) self-perceived level of salt consumption and (2) perceived importance of reducing salt intake. Responses were recoded so that healthier attitudes corresponded to higher values, normalized to a 0–1 scale, and averaged. The resulting Attitude Index was then dichotomized into favourable versus unfavourable attitude and used as a binary outcome in the regression models. The Attitude Index was used as a pragmatic composite indicator for analytical purposes rather than as a standalone validated psychometric scale.
– Salt-related behaviours related to salt use: Self-reported practices aimed at reducing salt consumption. Three items were used: (1) frequency of adding salt or salty sauces to food at the table, (2) adding salt during cooking, and (3) consuming salty processed/ready-made salty foods. All items were recoded so that healthier behaviour (lower salt use) corresponded to higher values, normalized to a 0–1 scale, and averaged. The resulting behaviour index was then dichotomized into favourable vs. unfavourable behaviour and used as a binary outcome in the regression models.

Thus, all outcomes were analysed as binary variables for logistic regression.

### 2.9. Statistical Analysis

All collected data were initially organized and validated in Microsoft Excel and then analyzed using SPSS software (version 24.0 for Windows), specifically the Complex Samples module, which accounted for the survey’s multistage cluster design. Sampling weights were applied to correct for unequal selection probabilities and response rates, with stratification based on region and age group, and primary sampling units defined at the level of primary healthcare centers. Standard errors and 95% confidence intervals were calculated using the Taylor series linearization method.

Continuous variables are presented as means ± standard deviations and categorical variables as percentages with 95% confidence intervals. Comparisons of means between two groups were performed using Student’s *t*-test, and between multiple groups using one-way ANOVA. Differences in categorical distributions were assessed using Pearson’s chi-squared test (χ^2^). A *p*-value < 0.05 was considered statistically significant.

To assess associations between socio-demographic characteristics and dietary patterns (fruit and vegetable consumption and salt-related outcomes), we used binary logistic regression. The dependent variables were grouped into two thematic blocks: (1) adequate fruit and vegetable consumption (≥5 servings/day vs. <5 servings/day) and (2) salt-related knowledge, attitudes, and behaviours (each analysed as a binary outcome as described above). Pearson’s chi-squared test was used for descriptive bivariate comparisons, whereas logistic regression models were used to estimate the direction and magnitude of associations as odds ratios (ORs) with 95% confidence intervals (95% CIs).

Rather than fitting a single model including all socio-demographic predictors simultaneously, we used prespecified minimally adjusted logistic regression models for each exposure–outcome pair. In each model, one exposure of interest (sex, age group, ethnicity, administrative unit, marital status, education level, occupation, BMI category, smoking status, HED, and, for salt-related outcomes, self-reported hypertension status) was entered as the main independent variable, and the dietary indicator (adequate fruit and vegetable consumption; salt-related knowledge; salt-related attitude; salt-related behaviour) was entered as the dependent variable.

For each exposure, models were adjusted only for a priori confounders identified based on assumed causal ordering, that is, factors considered likely to precede both the exposure and the outcome and unlikely to lie on the causal pathway. Odds ratios (ORs) and 95% confidence intervals (95% CIs) are reported. Full results of these minimally adjusted models, including the set of a priori confounders used for each exposure–outcome combination (shown in the “Adjusted for” column), are presented in Supplementary Table S2.

To visualize the prevalence of adequate fruit and vegetable consumption across different administrative units of Kazakhstan, a map was created based on survey data using the free online tool Datawrapper (https://www.datawrapper.de/, accessed on 17 October 2025).

## 3. Results

### 3.1. Demographic Characteristics

The study sample consisted of 6 720 participants aged 18–69 years (mean age = 40.8 ± 13.9 years; median = 39 years), of whom 3365 (50.1%) were men and 3355 (49.9%) were women. Turkic ethnicities accounted for 70.4% of respondents, whereas Slavic ethnicities comprised 24.7%. Among participants, 64.2% had higher or postgraduate education, 67.2% of the participants were married, 55.9% had overweight or obesity, 19.1% were currently tobacco users and 8.2% reported HED (Table 1).

### 3.2. Fruits and Vegetables

**Figure 1:**
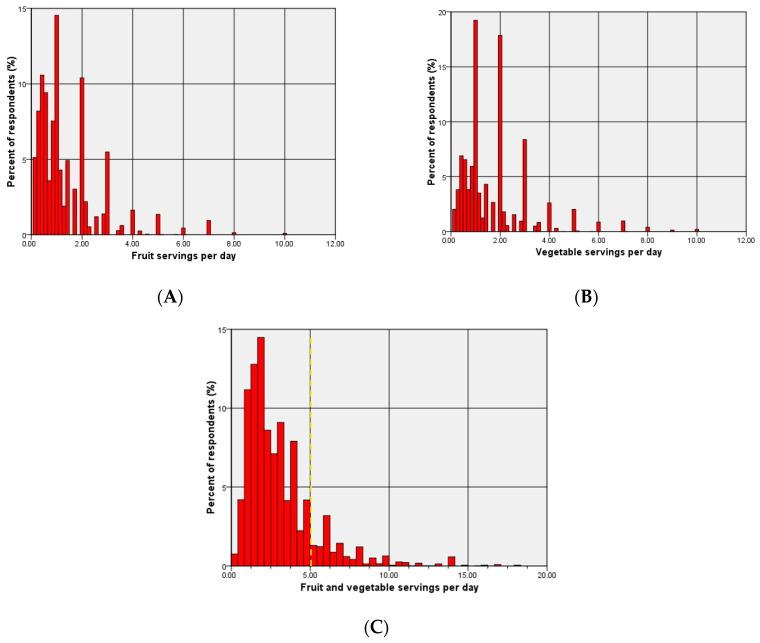
Distribution of fruit and vegetable consumption in adults. (**A**) Fruit servings per day. (**B**) Vegetable servings per day. (**C**) Total fruit + vegetable servings per day. The dashed vertical line marks the WHO recommendation of ≥5 servings/day.

Figure 1 shows the distributions of daily fruit, vegetable, and total fruit and vegetable consumption; all were strongly right-skewed, with most respondents consuming ≤ 3 servings/day. Daily consumption was reported by 33.0% for fruits and 50.6% for vegetables. Among 6003 respondents with available data on fruit and vegetable consumption, 15.7% (n = 940; 95% CI 14.8–16.7) achieved the WHO recommendation of ≥5 total servings per day, while 84.3% consumed fewer than five servings per day.

### 3.3. Gender Differences

**Figure 2:**
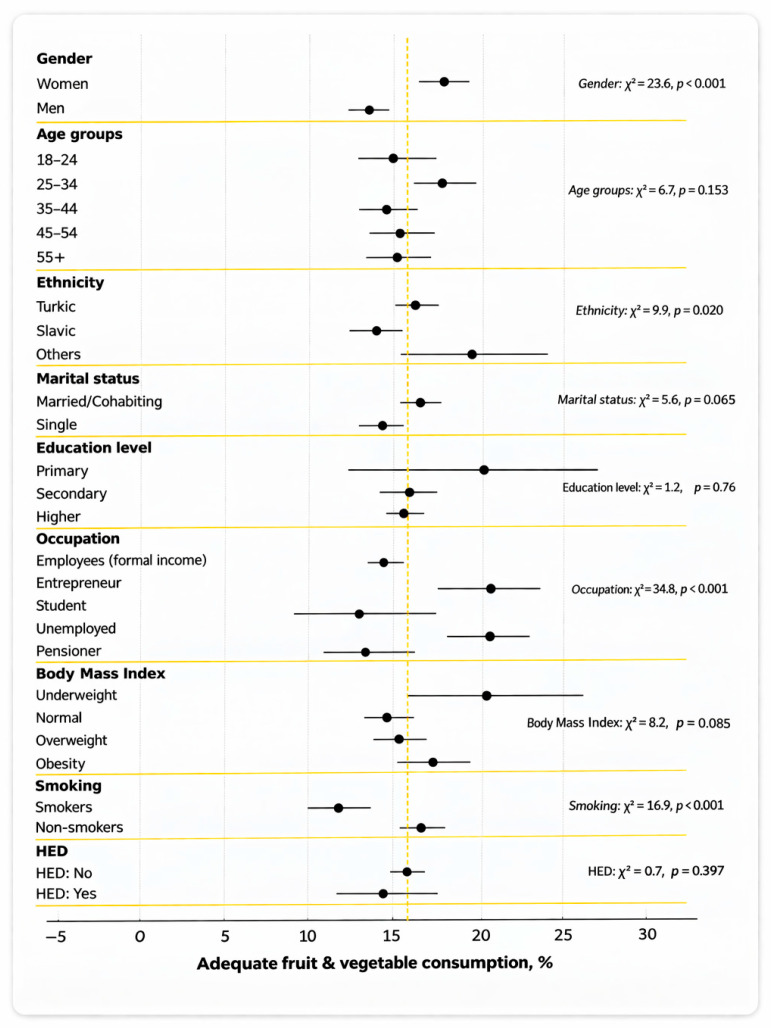
Adequate fruit and vegetable consumption (≥5/day) by sociodemographic and behavioural group. Horizontal bars represent 95% confidence intervals; the dashed vertical line indicates the overall weighted prevalence.

Adequate fruit and vegetable consumption was generally uncommon in both sexes: only 17.9% of women (95% CI 16.6–19.3) and 13.4% of men (95% CI 12.2–14.6) met the recommendations (Figure 2). Women also reported a slightly higher mean combined fruit and vegetable consumption (3.2 servings per day) compared with men (2.9 servings per day) (Table S1).

### 3.4. Age Characteristics

The proportion of participants with adequate consumption did not show a clear age trend. The highest proportion was observed in the 25–34 age group (17.7%, 95% CI: 15.8–19.8), and the lowest in the 35–44 age group (14.5%, 95% CI: 12.7–16.4). No statistically significant differences were found between age groups (χ^2^ = 6.7, *p* = 0.153).

### 3.5. Ethnic Differences

Turkic ethnicities had a higher prevalence of adequate fruit and vegetable consumption than Slavic ethnicities: 16.1% (95% CI 15.0–17.3) vs. 13.7% (95% CI 12.0–15.4); χ^2^ = 9.9, *p* < 0.020. Respondents from other ethnic groups showed the highest apparent prevalence, 19.6% (95% CI 15.4–24.4), but this estimate is less precise due to the smaller sample size in this category (n = 323).

### 3.6. Socio-Economic Factors

The percentage of participants with adequate fruit and vegetable consumption was 16.4% among married and cohabiting individuals and 14.1% among single individuals; however, this difference was not statistically significant (χ^2^ = 5.9, *p* = 0.065).

The association between education level and fruit and vegetable consumption was not statistically significant (χ^2^ = 1.2, *p* = 0.763). However, among individuals with primary education, the level of adequate consumption was formally higher (20.0%), but with a wide 95% CI, which may be related to the small sample size (n = 83).

Entrepreneurs showed the highest level of adequate fruit and vegetable consumption (21.1%), while pensioners and students showed the lowest (13.3% and 12.7%, respectively). The differences were statistically significant (χ^2^ = 34.8, *p* < 0.001).

### 3.7. Body Mass Index and Risk Behaviours

Across BMI categories, adequate fruit and vegetable consumption showed no clear monotonic trend, with proportions clustered within ≈15–20% (e.g., 14.7% at normal BMI vs. 17.2% in obesity). Among smokers, the proportion of individuals with adequate fruit and vegetable consumption was only 11.7% (95% CI: 10.0–13.7), while among non-smokers this indicator was significantly higher—16.6% (95% CI: 15.6–17.7). The differences were statistically significant (*p* = 0.001). The average fruit consumption among smokers was also lower—1.1 servings per day versus 1.4 among non-smokers (Table S1). No association between HED and the level of consumption was found (χ^2^ = 0.719, *p* = 0.397).

### 3.8. Regional Differences

**Figure 3:**
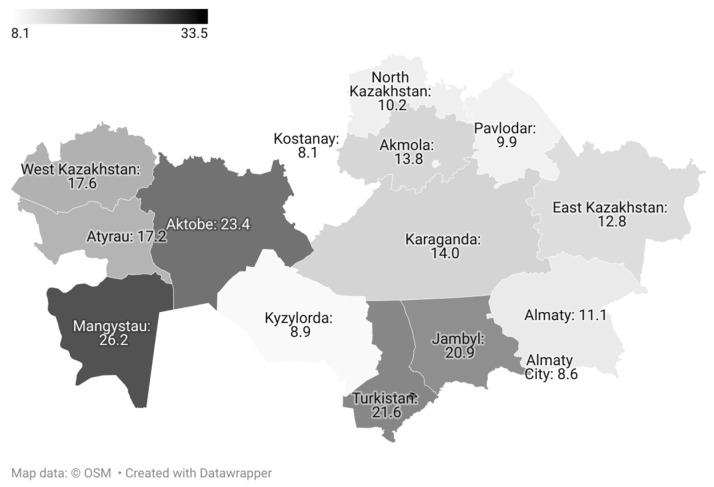
Percentage of adults with adequate fruit and vegetable consumption by administrative units of Kazakhstan.

The prevalence of adequate fruit and vegetable consumption varied markedly across regions. It was lowest in the northern regions—Kostanay, Pavlodar, and North Kazakhstan—and in the cities of Astana (9.4%) and Almaty (8.6%), and highest in Shymkent (33.5%), Turkestan (21.6%), and Mangystau (26.2%) (Figure 3).

In minimally adjusted logistic regression models, where each sociodemographic variable was examined separately while controlling for pre-specified confounders, men had substantially lower odds of achieving adequate fruit and vegetable consumption compared with women (aOR = 0.72; 95% CI: 0.62–0.84) (Table S2).

No statistically significant associations between adequate consumption and age, ethnicity, marital status, or educational level were observed. By occupational group, higher odds of adequate consumption were found among entrepreneurs (aOR = 1.92; 95% CI: 1.30–2.83) and unemployed participants (aOR = 1.52; 95% CI: 1.06–2.17) compared with pensioners. Participants with normal weight and overweight had somewhat lower odds of adequate consumption than those with obesity (aOR = 0.81; 95% CI: 0.68–0.95 and aOR = 0.78; 95% CI: 0.66–0.91, respectively). Current smokers were also less likely to meet the recommended intake compared with non-smokers (aOR = 0.78; 95% CI: 0.63–0.97).

A pronounced inter-regional gradient was observed: compared with Shymkent, which was used as the reference category, all other regions showed lower odds of adequate consumption (aORs ranged from 0.16 to 0.65). The lowest odds were observed in Kostanay (aOR = 0.16; 95% CI: 0.10–0.26), Pavlodar (aOR = 0.20; 95% CI: 0.12–0.31), and North Kazakhstan (aOR = 0.20; 95% CI: 0.11–0.33) regions, as well as in the cities of Astana (aOR = 0.19; 95% CI: 0.13–0.28) and Almaty (aOR = 0.18; 95% CI: 0.12–0.26).

### 3.9. Salt-Related Outcomes

Descriptive distributions of salt-related knowledge, attitudes and behaviours by socio-demographic, behavioural and clinical subgroups are summarised in Table 2 and Supplementary Table S3.

### 3.10. Knowledge

**Figure 4:**
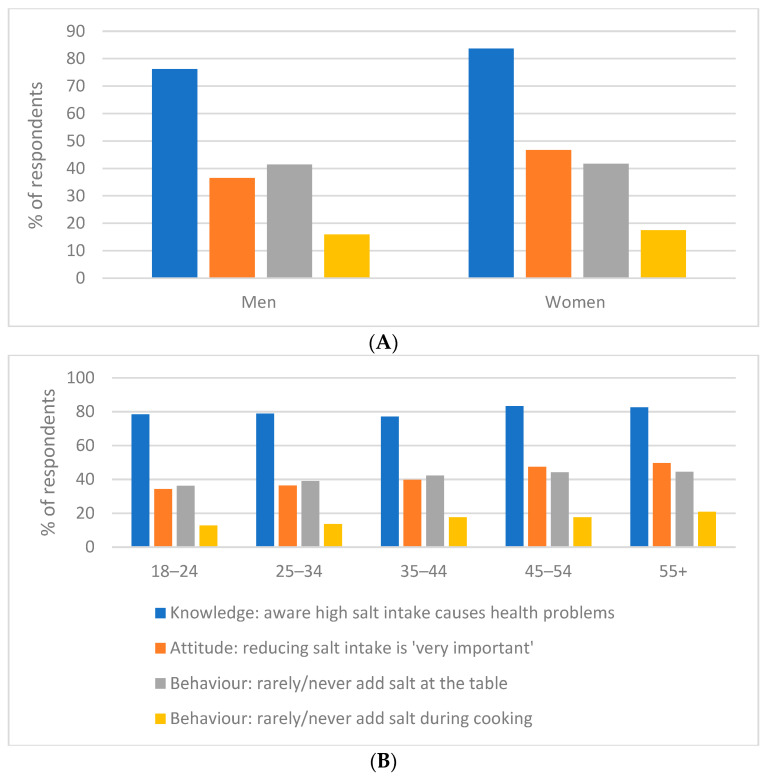
Gender (**A**) and age (**B**) differences in salt-related knowledge, attitudes, and behaviours among adults in Kazakhstan.

The majority of participants (about 80.6%) knew that excessive salt consumption can lead to serious health problems. Women were generally more aware than men (83.7% vs. 76.2%, *p* < 0.001) (Figure 4). The level of knowledge increased with the age of respondents and with higher education (*p* < 0.001). Significant differences were also observed by occupation and region. Pensioners turned out to be the most knowledgeable group (about 84% knew about the harm of excess salt), whereas students had the lowest rate (≈75%). The highest proportion of informed respondents was recorded in Shymkent (90.2%), while the lowest values were in Aktobe (60.6%) and Almaty (70.8%) regions. In addition, individuals diagnosed with arterial hypertension demonstrated a higher level of knowledge (85.9% knew about salt-related risks, *p* < 0.001) compared to other groups.

**Figure 5:**
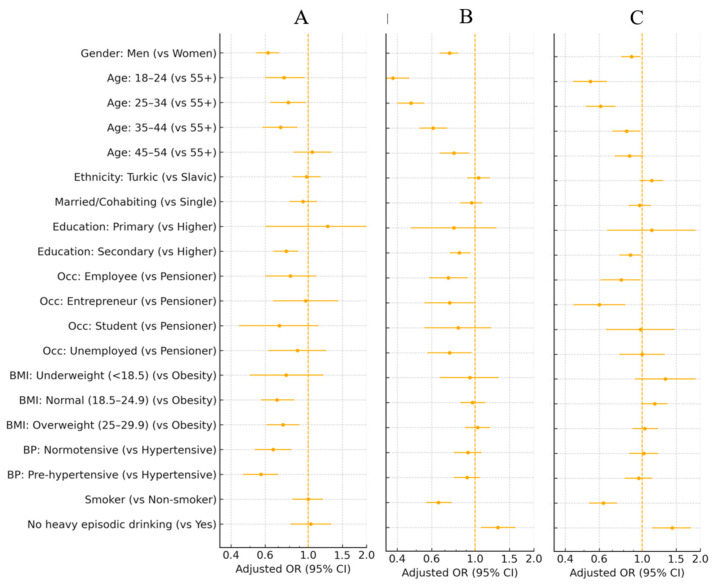
Minimally adjusted odds ratios for salt-related knowledge (**A**), attitudes (**B**), and behaviours (**C**) by socio-demographic characteristics. Points represent adjusted odds ratios, horizontal bars represent 95% confidence intervals, and the vertical reference line indicates OR = 1.0.

In minimally adjusted logistic regression models (Figure 5A; Table S2), men had significantly lower odds than women of acknowledging that high salt intake can cause serious health problems (aOR 0.62; 95% CI 0.54–0.71). Adults aged 18–44 years were also less likely to report good knowledge compared to those aged 55 years and older, and participants with secondary versus higher education had reduced odds of awareness. Respondents with normal weight or overweight and those without diagnosed hypertension were less likely to report adequate knowledge than individuals with obesity or hypertension. A pronounced regional gradient was also evident: compared with Shymkent city, which had the highest prevalence of awareness, most other regions showed substantially lower odds of good knowledge (adjusted ORs generally between 0.16 and 0.55), with the largest deficits observed in Aktobe, Kyzylorda and Almaty regions. Only East Kazakhstan did not differ significantly from Shymkent after adjustment (aOR 0.82; 95% CI 0.52–1.30). Ethnicity, marital status, occupational group, smoking status and heavy episodic drinking showed no clear associations with salt-related knowledge after adjustment for confounders.

### 3.11. Attitude

Despite the high level of awareness, less than half of respondents (41.7%) considered reducing salt intake to be very important for health. Women attached greater importance to this than men (46.7% vs. 36.5%, *p* < 0.001) (Figure 5). With age, the importance of salt reduction in the eyes of respondents noticeably increased: among young people aged 18–24, only about 34% named salt reduction as a priority, while in the group over 55 years old almost 50% thought so (*p* < 0.001) (Figure 5). Highly educated participants and pensioners also significantly more often noted the importance of reducing salt intake (*p* < 0.001 compared to less educated and working respondents, respectively). Gender differences in self-assessment of salt intake were statistically significant but minor in magnitude. In the youth group (18–24 years), 16.5% of respondents considered their salt intake high, whereas among those aged 55 and older only 9.5% thought so. With age, the percentage of those who assessed their intake as “low” increased (χ^2^ = 81.7; *p* < 0.001). Participants with primary education more often than others believed that they consumed a lot of salt (25.8% vs. 12.9% among those with higher education; χ^2^ = 10.5; *p* = 0.032). By occupation, the highest proportion of high self-assessments was recorded among students and the unemployed (≈15%), and the lowest among pensioners (7.2%; χ^2^ = 59.5; *p* < 0.001). Respondents who smoked or abused alcohol more often considered their salt intake high and less often low (both trends *p* < 0.01). Among those with hypertension, the proportion assessing their intake as “high” was comparable to others (~13%), but they significantly more often classified themselves in the low consumption category (26.3% vs. ~21% in other groups; χ^2^ = 17.3; *p* = 0.002), which was reflected in a reduced percentage of “normal” responses. Clear ethnic and regional differences in self-assessment emerged: most often, high salt intake was reported by residents of southern regions (Shymkent, Kyzylorda, and Turkestan regions ~18–19%). The lowest proportion of such responses was noted in Pavlodar and Almaty regions (≈9–10%), where, on the contrary, answers of “normal” intake predominated (up to 74% of respondents).

In minimally adjusted logistic regression models (Figure 5B; Supplementary Table S2), men had lower odds than women of agreeing that salt consumption should be reduced (aOR 0.74; 95% CI 0.66–0.82). Younger adults were consistently less likely to support salt reduction than those aged 55 years and older, with adjusted odds ratios of 0.38 (18–24 years), 0.47 (25–34 years), 0.61 (35–44 years) and 0.78 (45–54 years). Participants with secondary education also showed slightly weaker support compared with those with higher education (aOR 0.83; 95% CI 0.74–0.95), whereas primary education did not differ significantly from higher education. Employees with formal income, entrepreneurs and unemployed respondents had lower odds of a favourable attitude than pensioners (aOR 0.73, 0.74 and 0.74, respectively). Current smokers were less likely than non-smokers to endorse salt reduction (aOR 0.65; 95% CI 0.56–0.76), while respondents without heavy episodic drinking were more likely to report a positive attitude (aOR 1.31; 95% CI 1.07–1.61). Marked regional variation was observed: compared with Shymkent city, odds of supporting salt reduction were higher in Aktobe region (aOR 1.81; 95% CI 1.30–2.50), but lower in several other regions, including Astana city, Almaty city, Almaty, West Kazakhstan, Zhambyl, Karaganda, Kostanay, Mangystau, Pavlodar, North Kazakhstan and East Kazakhstan (all *p* < 0.05).

### 3.12. Behaviour

The most common practice among respondents was adding salt during cooking (64.6% reported such behaviour). Salt was added at the table much less frequently (39.6%), while a relatively small proportion of participants regularly consumed salted processed foods (about 29%—“often” or “very often”). Almost one-third of respondents reported that they sometimes consumed such products, while 37.3% tried to eat them rarely or never. Men consumed industrially salted foods somewhat more often than women (31.3% vs. 27.4%; *p* = 0.002) (Figure 5). With increasing age, behaviour changed: the proportion of those who often added salt at the table decreased, whereas the habit of salting food during cooking became more common (*p* < 0.001) (Figure 5). People with lower education were more likely to add salt during cooking and more often consumed salty foods (*p* < 0.01 compared to more educated individuals). Among students and the unemployed, the highest indicators of “salt-related” behaviour were recorded (about 38–42% of them often salted food and/or ate a lot of salty products), while pensioners demonstrated the most moderate habits. Respondents who smoked or consumed large amounts of alcohol were significantly more likely to salt food during cooking (up to 75% in these subgroups) and regularly ate salty foods (up to 39%; *p* < 0.001 for all comparisons). People with arterial hypertension were more likely to add salt during cooking (49.6% vs. lower proportions among others, *p* < 0.001), but in other aspects their behaviour did not differ from the rest of the respondents. Ethno-cultural and regional differences also emerged: the most “salty” habits were recorded among Uighurs and residents of southern regions, while the lowest were in Pavlodar and Almaty regions (*p* < 0.001).

In minimally adjusted logistic regression models (Figure 5C; Supplementary Table S2), men had slightly lower odds than women of reporting favourable salt-related behaviours (aOR 0.88; 95% CI 0.78–0.98). Age showed a clear gradient: compared with adults aged 55 years and older, younger participants were less likely to adhere to recommended, salt-moderate habits, with adjusted odds ratios of 0.54 (18–24 years), 0.61 (25–34 years) and 0.83 (35–44 years), while the 45–54 age group did not differ significantly. Participants with secondary education were also less likely to report healthy behaviours than those with higher education (aOR 0.87; 95% CI 0.76–0.99), whereas primary education did not show a clear association. Among occupational groups, employees with formal income and especially entrepreneurs had lower odds of favourable behaviour compared with pensioners (aOR 0.78; 95% CI 0.61–0.98 and 0.60; 95% CI 0.44–0.82, respectively), while students and unemployed respondents did not differ from pensioners. Current smokers were less likely than non-smokers to follow recommended practices (aOR 0.63; 95% CI 0.53–0.74), whereas respondents without heavy episodic drinking were more likely to report healthy behaviour (aOR 1.43; 95% CI 1.13–1.79). Regional differences were also evident: compared with Shymkent city, odds of favourable behaviour were higher in Astana and Aktobe regions (aOR 1.46 and 1.49, respectively), but lower in Almaty and East Kazakhstan regions (aOR 0.43 and 0.58, both *p* < 0.01); other regions did not differ materially from Shymkent. Ethnicity, marital status, body mass index and hypertension status were not significantly associated with salt-related behaviours after adjustment for confounders.

## 4. Discussion

The discovery of a low level of adequate fruit and vegetable consumption among the adult population of Kazakhstan indicates a large-scale public health problem. However, this is not surprising, as numerous studies show that low fruit and vegetable consumption is a global phenomenon [26].

According to a multicenter analysis in 28 low- and middle-income countries, more than 80% of adults did not meet WHO recommendations, while similar trends are observed in high-income countries [7,27]. A lack of fruits and vegetables in the diet has been recognized as a risk factor for more than 6.7 million deaths per year [28].

Globally, more than three-quarters of the adult population (77–78%) do not meet the recommended intake. Average fruit and vegetable consumption equals 1.4 and 1.7 portions per day respectively, which is below the threshold of 5 portions and even significantly lower than the average indicators in low- and middle-income countries (LMICs), where the total intake amounts to 3 to 4 portions per day [29]. At the same time, in Kazakhstan the threshold is exceeded only by one sixth of the population.

Equally alarming are the data demonstrating a pronounced gap between awareness of the harms of excessive salt intake and actual behaviour. Although about 80.6% of respondents acknowledged the negative consequences of excessive salt consumption, only 41.7% considered its limitation extremely important, and an even smaller proportion followed the relevant recommendations. Such inconsistency between cognitive level and behavioural attitudes has been repeatedly documented in international studies [30]. In a number of countries, most respondents recognize the need to reduce salt consumption, but in practice rarely abandon their habitual salty dishes or choose products with reduced sodium content [31,32]. This underlines that awareness alone is not a sufficient condition for changing everyday behaviour. In the absence of clear product labeling, subsidies for alternatives, and the social normalization of “low-salt behaviour”, knowledge remains declarative, without being transformed into conscious daily practice [33].

International studies confirm that excessive salt intake is one of the most persistent behavioural risk factors in countries with transition economies [34]. At the same time, excessive salt consumption in the Central Asian region may be associated not only with the habit of adding salt to food, but also with a high proportion of hidden salt in semi-finished products, bread, and traditional dishes.

Thus, in combination with insufficient fruit and vegetable consumption, high salt intake forms a double burden, where the diet is poor in protective components but oversaturated with pro-atherogenic ones [35,36]. This is a typical picture for countries undergoing epidemiological transition [37], and requires a comprehensive strategy with an emphasis not only on raising awareness but also on changing the structure of consumption at the level of the entire food system. Our findings should also be interpreted within the scope of the present study, which focused on fruit, vegetable, and salt-related behaviours rather than on total energy intake or overall dietary patterns.

Therefore, in discussing the results of our study, it is necessary to look through the prism of systemic barriers, namely demographic, socio-economic, cultural, and behavioural ones.

### 4.1. Gender and Age Differences

Women in our study more consistently reported healthier eating patterns than men, including a higher likelihood of meeting recommended fruit and vegetable consumption. This pattern mirrors what is described in international adult populations, where women generally show greater adherence to nutrition-related recommendations and a more health-oriented food choice profile [38]. In our data, this difference between women and men persisted even after accounting for socioeconomic and behavioural factors, suggesting that it is not solely a function of income, education, or awareness, but may reflect more stable gendered norms around food and health.

Findings in younger adults also align with published evidence. The EAT study, which followed individuals in early adulthood, showed that fruit and vegetable consumption remained very low several years after adolescence, and highlighted taste preference, home availability of fresh produce, and perceived time constraints as key factors shaping diet in this age group [39]. We observed a similar pattern: younger adults did not necessarily consume dramatically fewer fruits and vegetables than older adults, but they were less likely to reach an intake level that would be considered nutritionally adequate. This supports the idea that early eating habits tend to persist into adulthood unless the environment changes in a way that facilitates healthier choices, for example through affordability and convenient access [40].

Gender and age differences were also apparent in salt-related behaviour. Women were more likely than men to report actively limiting salt in their diet, and this tendency remained after adjustment for socioeconomic status and risk awareness. Younger adults, in contrast, were the least inclined to reduce salt, even when they recognized health risks. This is consistent with the interpretation that immediate taste preference and low perceived vulnerability dominate decision-making at younger ages, whereas with increasing age (and with accumulation of clinical experience such as diagnosis of hypertension) people become more willing to modify behaviour [30].

### 4.2. Ethnic Differences

Apparent differences in fruit and vegetable consumption were initially observed between Turkic and Slavic groups, suggesting a possible role of ethnocultural dietary patterns. However, once demographic and socioeconomic factors were accounted for, ethnicity itself was no longer an independent predictor of adequate intake. This aligns with international evidence indicating that, in multiethnic settings, dietary differences between ethnic and racial groups often reflect broader structural and environmental inequalities rather than inherent cultural preferences [41].

In Kazakhstan, everyday food practices are progressively converging across ethnic groups, which further reduces the explanatory power of ethnicity alone [13]. We therefore consider that future work on ethnic patterns in diet should incorporate qualitative methods—for example, focus groups and in-depth interviews—to capture household norms, intergenerational transmission, and food environments in a multiethnic context [13].

### 4.3. Socio-Economic Factors

Our analysis showed that entrepreneurs demonstrate the highest level of adequate fruit and vegetable consumption, which is several times higher than that of pensioners and students. This pattern may reflect broader occupational and socio-economic differences, although actual income was not measured and the role of purchasing power cannot be assessed directly in our data. Previous studies suggest that socio-economic status may play an important role in shaping dietary habits [42,43], and that under conditions of limited resources, people may prioritize cheaper and more familiar foods over a healthier diet [44].

However, these same entrepreneurs demonstrate worse behavioural habits regarding salt consumption (41.9% always/often add salt to food at the table, 32.1% regularly consume salty processed foods), which may be related to lifestyle characteristics rather than socio-economic status. In turn, this corresponds to the “price–time” model, where high cost and lack of time lead to the choice of less healthy but more convenient food [45].

Willingness to reduce salt consumption differed across educational levels. People with higher education are more likely to report being ready to limit salt intake compared to those with primary education. This indicates a strong association between education level and adherence to recommendations for reducing salt consumption. This pattern is consistent with international data, where education level is a stable predictor of behavioural compliance with salt guidelines [46].

At the same time, the data are alarming, showing that even among the wealthiest and most educated respondents, more than half do not apply even basic salt reduction strategies. This speaks not so much of a lack of resources as of weak institutional support and the absence of continuous education formed from childhood [47]. Behaviour remains more of a reaction than a sustainable norm.

### 4.4. Regional Differences

Regional disparities in fruit and vegetable consumption behaviour in our data turned out to be very sharp. Northern regions and major cities (Astana, Almaty) show the lowest (no more than 10%) level of adequate consumption, whereas Shymkent and Turkestan are in the range of 25–35%.

Such regional patterns are not unique to Kazakhstan. Agroeconomic and infrastructural differences, climate, and subsidy policies affect the availability of fresh fruits and vegetables [48]. For example, having home gardens increases consumption and reduces BMI, while geographic consumption clusters correlate with availability, income, and cultural traditions.

In Kazakhstan’s wealthier cities, lower fruit and vegetable consumption, in our view, is not due to overall availability, but rather to a number of local factors. First, the urban diet more often includes fast food and processed products. Second, high employment, especially among working youth and migrants, reduces attention to diet. Third, even when products are available on the market, quality fruits and vegetables are often expensive, creating barriers to regular consumption [48].

Interestingly, in terms of behavioural characteristics related to salt, the picture turned out to be the opposite. In Almaty, Astana, and Karaganda, the percentage of people actively limiting salt in their diet (i.e., following ≥ 2 behavioural measures) was significantly higher (31–34%) than in Turkestan, Shymkent, and Zhambyl region (19–22%). This paradox—higher fruit and vegetable consumption but lower salt self-regulation—is especially pronounced in the southern regions. However, this phenomenon is not unique. In a number of countries with strong regional cultural specifics, cases are observed where positive habits in some components are combined with risky ones in others [49].

It is not enough to stimulate only fruit and vegetable consumption; it is also necessary to reduce habitual salt consumption, especially hidden salt. For the Republic of Kazakhstan, this is particularly relevant in the southern regions, where the high prevalence of hypertension is already combined with high salt load, despite an outwardly “healthy” diet. Salt reduction policies should not be universal but tailored to the everyday gastronomy of specific regions.

Our findings also have practical relevance, as they help identify the population groups and regions in which unhealthy dietary patterns are more common. This may support the development of more targeted public health actions, including educational initiatives, regionally adapted nutrition programmes, and broader strategies aimed at improving access to healthier foods and reducing excess salt consumption. At the same time, these patterns are likely to reflect a combination of contextual influences, including food availability and affordability, urban food environments, and cultural dietary practices, all of which should be examined more directly in future research.

### 4.5. BMI, Smoking, and Alcohol

Our data confirm that BMI is not associated with adequate fruit and vegetable consumption, where the level ranged from 16.3% among individuals with normal body weight to 19.0% among those with underweight, with no statistically significant differences found. This is consistent with international studies, where no consensus exists on this matter [50]. Higher fruit and vegetable consumption has been associated with modest reductions in BMI in some populations, particularly among individuals with obesity; however, findings are inconsistent and appear to vary with physical activity, overall energy balance, and broader dietary patterns [51].

Smoking turned out to be a significant marker of unhealthy dietary behaviour: in our sample, only 12.5% of smokers consumed an adequate amount of fruits and vegetables compared to 17.4% among nonsmokers. These findings are consistent with international research showing that smokers systematically consume less plant-based food. Possible reasons include both biobehavioural features (clustering of harmful habits) and changes in taste perception caused by tobacco exposure. In turn, higher fruit intake is associated with successful attempts to quit smoking [52].

The relationship between alcohol consumption and diet is less straightforward. In our sample, moderate alcohol consumption did not affect the frequency of adequate fruit and vegetable intake but was associated with less favourable levels of knowledge and behaviour regarding salt. This partially aligns with studies showing that people with higher consumption of fruits and vegetables on average drink less alcohol and smoke less, although the correlations are not always stable and vary across countries [53].

Although BMI itself was not associated with fruit and vegetable consumption in our study, smoking demonstrated a consistent link with unhealthy diet. Alcohol appears more as a behavioural marker than a direct determinant.

Smokers and individuals who abuse alcohol were also less likely to adhere to salt reduction recommendations. This is consistent with studies showing that high sodium intake is more common among people who regularly consume alcohol and smoke. It has been found that smokers and heavy alcohol consumers are 2.5–3 times more likely to prefer salty food compared to nonsmokers and moderate drinkers [54,55,56]. Such preference may be driven both by physiological changes in taste perception and by cultural norms of consuming salty snacks with alcohol [43,44,45].

The findings highlight the need to move from isolated information campaigns to comprehensive and sustainable strategies for reducing salt consumption at the population level. Raising awareness alone is insufficient—it is necessary to simultaneously foster sustainable attitudes, change behavioural norms, and create an external environment that supports healthy choices. As noted by S. Nurmilah et al. [57], behavioural strategies such as gradual salt reduction, reminders, and substitution with less salty alternatives have high potential but are rarely implemented in practice.

Meanwhile, successful international experience demonstrates the effectiveness of systemic measures. In the United Kingdom, a program of category-specific sodium targets with independent monitoring and industry reporting reduced population salt intake by ≈16% and lowered the sodium density of foods within a decade [58]. In Europe, modelling suggests that salt-reformulation scenarios could translate into substantial cardiovascular benefits—for example, a 10–23% reduction in stroke prevalence with a 30% salt reduction in Finland and Poland [32]. In Japan, sustained reformulation and standards in public catering supported a long-term decline in mean salt intake from ~14.5 g/day in the 1970s to ~9.5 g/day by 2017 [59].

Importantly, retrospective policy analyses from four low- and middle-income countries show that different policy mixes can be effective when matched to context. South Africa adopted legislated maximum sodium limits for staple categories (e.g., bread), driving measurable reductions in product sodium content; Argentina also used legislation, whereas Mongolia and Vietnam pursued voluntary targets with industry engagement and public-sector standards. Across settings, success depended on clear, time-bound category targets (voluntary or mandatory), multisector coordination (health, education, industry), transparent monitoring (product audits, repeat intake surveys), and stable funding and technical support; common barriers were limited local data, capacity constraints, and leadership turnover [37].

Taken together, these findings point to the need for a coordinated multisectoral response in Kazakhstan, with the main practical implications summarized in the Conclusion.

## 5. Conclusions

Adults in Kazakhstan show insufficient fruit and vegetable consumption and suboptimal salt-related knowledge, attitudes, and behaviours. Less favourable patterns were observed more often among men, younger adults, smokers, respondents with lower educational attainment, and residents of several administrative units, indicating the need for targeted and geographically adapted interventions.

These findings support the development of a coordinated, multisectoral policy response. Priority actions may include front-of-pack labeling, category-specific sodium reformulation targets, nutrition standards in public procurement settings, and support for lower-salt food environments, including appropriate use of potassium-enriched salt substitutes and default no-added-salt practices in food service. Such measures should be supported by a national monitoring system incorporating periodic sodium surveillance, product-level monitoring, and clear implementation targets. Particular attention should be paid to equity, so that healthier dietary options are accessible and affordable across regions.

## 6. Strengths and Limitations of This Study

This study has several strengths. The use of a multistage stratified sampling with cluster selection ensured the reliable representativeness of the data for all 17 administrative units of Kazakhstan. This study included participants of different age, social, and ethnic groups, which increased the statistical power and allowed extrapolation of the results to the national level. The application of standardized WHO tools (the STEPS questionnaire) ensured comparability with international studies and high methodological quality. At the same time, this study has certain limitations. Its cross-sectional design does not allow for conclusions about causal relationships, and all identified associations should be interpreted as correlational. Data on dietary behaviour and fruit/vegetable intake, as well as salt-related indicators, were based on self-reports by respondents, which may be subject to memory errors and social desirability bias. In particular, these measures do not provide an objective assessment of actual salt intake. The use of more objective methods (e.g., food diaries or biomarkers) could have increased the accuracy of the assessment. Another limitation is that dietary patterns may vary by season and region in Kazakhstan, so our findings should be interpreted as a nationally representative snapshot during the survey period rather than a full reflection of year-round dietary behaviour. Because our analyses involve multiple logistic models, based on prespecified minimally adjusted sets of covariates, the likelihood of chance findings due to multiple comparisons increases, so individual associations—especially secondary and subgroup analyses—should be interpreted with caution. Finally, recruitment of participants through primary healthcare may lead to selection bias, since individuals seeking medical care may differ in health-related behaviours from the general population.

## Figures and Tables

**Table 1:** Socio-demographic and behavioural characteristics of the study population (n = 6720).

Variable	n	%
**Total**	6720	100.0
**Gender**		
Men	3365	50.1
Women	3355	49.9
**Age groups**		
18–24	919	13.7
25–34	1641	24.4
35–44	1525	22.7
45–54	1268	18.9
55+	1367	20.3
**Ethnicity**		
Turkic	4730	70.4
Slavic	1660	24.7
Others	323	4.8
No answer	7	0.1
**Marital status**		
Married/cohabiting	4519	67.2
Single	2192	32.6
No answer	9	0.1
**Education level**		
Primary education	83	1.2
Secondary education	2207	32.8
Higher education	4316	64.2
No answer	114	1.7
**Occupation**		
Employees (formal income)	4307	64.1
Entrepreneur	552	8.2
Student	291	4.3
Unemployed	893	13.3
Pensioner	648	9.6
No answer	29	0.4
**Body Mass Index**		
Underweight (<18.5 kg/m^2^)	201	3.0
Normal (18.5–24.99 kg/m^2^)	2593	38.6
Overweight (25.0–29.9 kg/m^2^)	2387	35.5
Obesity (≥30.0 kg/m^2^)	1374	20.4
No answer	165	2.5
**Hypertension status**		
Normotensive	2256	33.6
Pre-hypertensive	2358	35.1
Hypertensive	2106	31.3
**Smoking**		
Smokers	1284	19.1
Non-smokers	5436	80.9
**HED**		
No	6168	91.8
Yes	552	8.2
**Region**		
Astana city	448	6.7
Almaty city	560	8.3
Akmola	336	5.0
Aktobe	336	5.0
Almaty region	560	8.3
Atyrau	336	5.0
West Kazakhstan	224	3.3
Zhambyl	448	6.7
Karaganda	448	6.7
Kostanay	336	5.0
Kyzylorda	336	5.0
Mangystau	336	5.0
Turkestan	560	8.3
Pavlodar	336	5.0
North Kazakhstan	224	3.3
East Kazakhstan	448	6.7
Shymkent city	448	6.7

**Table 2:** Key indicators of salt-related knowledge, attitudes, and behaviours: prevalence (%) and 95% CI by sociodemographic, behavioural, and clinical subgroups. Full category breakdown and χ^2^ tests are provided in Supplementary Table S3.

Subgroup	Knowledge: Aware of Harm	Attitude: Self-Rated High	Attitude: Reduction Very Important	Behaviour: Salt at Table	Behaviour: Salt During Cooking	Behaviour: Salty Processed Foods
**Total**	80.6 (79.6–81.5)	13.7 (12.9–14.5)	38.3 (37.2–39.5)	39.9 (38.8–41.1)	63.2 (62.0–64.3)	30.2 (29.1–31.3)
**Gender**						
Men	78.0 (76.6–79.4)	13.8 (12.7–15.0)	33.8 (32.2–35.4)	38.7 (37.0–40.3)	63.8 (62.1–65.4)	32.2 (30.7–33.8)
Women	83.2 (81.9–84.5)	13.6 (12.4–14.8)	43.0 (41.3–44.6)	41.2 (39.6–42.9)	62.6 (60.9–64.2)	28.2 (26.7–29.7)
**Age group**						
18–24	78.5 (75.8–81.1)	16.8 (14.4–19.3)	30.9 (28.0–33.9)	41.6 (38.4–44.8)	67.2 (64.2–70.2)	35.3 (32.2–38.4)
25–34	80.7 (78.8–82.6)	16.3 (14.6–18.2)	33.4 (31.1–35.7)	42.7 (40.3–45.1)	66.8 (64.5–69.0)	33.8 (31.6–36.1)
35–44	77.8 (75.7–79.9)	13.8 (12.1–15.6)	36.4 (34.0–38.8)	39.7 (37.3–42.2)	63.5 (61.0–65.9)	29.4 (27.2–31.8)
45–54	83.2 (81.1–85.3)	12.6 (10.9–14.5)	44.0 (41.3–46.7)	37.7 (35.1–40.4)	61.0 (58.3–63.6)	27.1 (24.7–29.6)
55+	82.7 (80.7–84.7)	9.4 (7.9–11.0)	46.2 (43.6–48.9)	37.8 (35.3–40.4)	57.9 (55.2–60.5)	26.1 (23.8–28.5)
**Education**						
Primary	88.0 (79.7–93.6)	24.1 (15.9–34.1)	54.2 (43.5–64.6)	54.2 (43.5–64.6)	53.0 (42.3–63.5)	34.9 (25.3–45.6)
Secondary	77.7 (75.9–79.4)	14.3 (12.9–15.8)	35.4 (33.5–37.4)	41.9 (39.9–44.0)	65.2 (63.1–67.1)	33.0 (31.1–35.0)
Higher	81.9 (80.7–83.0)	13.2 (12.2–14.2)	39.4 (37.9–40.9)	38.7 (37.2–40.2)	62.6 (61.2–64.1)	28.7 (27.4–30.1)
**Body mass index**						
Underweight (<18.5 kg/m^2^)	86.6 (81.3–90.7)	14.4 (10.1–19.8)	34.3 (28.0–41.1)	37.3 (30.8–44.1)	68.2 (61.5–74.3)	29.4 (23.4–35.9)
Normal (18.5–24.99 kg/m^2^)	83.0 (81.5–84.4)	14.4 (13.1–15.8)	38.7 (36.8–40.6)	40.5 (38.6–42.4)	66.7 (64.9–68.5)	31.0 (29.2–32.8)
Overweight (25.0–29.9 kg/m^2^)	81.3 (79.7–82.8)	13.4 (12.1–14.8)	38.5 (36.5–40.4)	41.9 (40.0–43.9)	63.0 (61.0–64.9)	29.6 (27.8–31.5)
Obesity (≥30.0 kg/m^2^)	74.2 (71.8–76.4)	12.4 (10.8–14.3)	39.2 (36.6–41.8)	36.3 (33.8–38.9)	56.6 (54.0–59.2)	29.3 (27.0–31.8)
**Hypertension status**						
Normotensive	82.6 (81.0–84.2)	14.1 (12.7–15.6)	35.0 (33.0–37.0)	41.2 (39.1–43.2)	64.7 (62.7–66.7)	30.4 (28.5–32.4)
Pre-hypertensive	77.7 (76.0–79.3)	13.1 (11.8–14.5)	35.4 (33.5–37.4)	40.1 (38.1–42.1)	63.5 (61.6–65.5)	28.9 (27.1–30.7)
Hypertensive	81.9 (80.2–83.5)	13.9 (12.5–15.5)	45.4 (43.3–47.6)	38.6 (36.5–40.7)	61.1 (59.0–63.2)	31.4 (29.4–33.4)
**Smoking status**						
Smokers	80.0 (77.7–82.1)	18.1 (16.1–20.3)	27.9 (25.5–30.4)	42.7 (40.0–45.4)	73.3 (70.8–75.7)	40.6 (37.9–43.3)
Non-smokers	80.7 (79.7–81.8)	12.6 (11.8–13.5)	40.8 (39.5–42.1)	39.3 (38.0–40.6)	60.8 (59.5–62.1)	27.7 (26.6–28.9)
**Heavy episodic drinking**						
No	80.7 (79.7–81.7)	13.4 (12.5–14.2)	39.0 (37.8–40.2)	39.5 (38.3–40.7)	62.4 (61.2–63.6)	29.3 (28.1–30.4)
Yes	79.3 (75.8–82.6)	17.4 (14.4–20.7)	30.8 (27.1–34.7)	44.7 (40.6–48.9)	71.6 (67.7–75.2)	40.6 (36.5–44.7)

Self-rated high = self-assessed salt consumption as very high/high; salt at table = adding salt at the table always/often; salt during cooking = adding salt during cooking always/often. HTN = self-reported hypertension diagnosis or elevated BP per STEPS definition, HED = heavy episodic drinking, CI = confidence interval. Subgroup counts are provided in Table 1 and Table S3.

## Data Availability

The original contributions presented in this study are included in the article/Supplementary Material. Further inquiries can be directed to the corresponding author.

## Supplementary Materials

The following supporting information can be downloaded at https://www.mdpi.com/article/10.3390/nu18071154/s1. Table S1. Fruit and vegetable intake by sociodemographic, behavioural, and regional subgroups: median (IQR) and mean (95% CI) daily servings. Table S2. Minimally adjusted logistic regression models for adequate fruit and vegetable intake and salt-related knowledge, attitudes and behaviours by socio-demographic characteristics. Table S3. Association between socio-demographic characteristics and knowledge, attitudes, and behaviours related to salt.

## Abbreviations

**Abbreviation**	**Definition**
WHO	World Health Organization
STEPS	WHO STEPwise approach to surveillance
PHC	Primary Health Care
PPE	Personal Protective Equipment
SOPs	Standard Operating Procedures
BMI	Body Mass Index
SBP	Systolic Blood Pressure
DBP	Diastolic Blood Pressure
LMICs	Low- and middle-income countries
EDTA	Ethylenediaminetetraacetic acid
KAB	Knowledge, Attitude, and Behaviour
HED	Heavy Episodic Drinking
OR	Odds Ratio
aOR	Adjusted Odds Ratio
CI	Confidence Interval
HTN	Hypertension
PSU	Primary Sampling Unit
COVID-19	Coronavirus disease 2019
NCDs	Non-communicable diseases
GDP	Gross Domestic Product
