# Supplementary material for: Association Between Socio-Demographic, Behavioural, and Health-Related Factors and Fruit, Vegetable, and Salt Consumption Among Adults Aged 18–69 Years in Kazakhstan: A Cross-Sectional Study
Source: Nutrients. 2026 Apr 3;18(7):1154. doi: 10.3390/nu18071154 (PMC13074515; doi:10.3390/nu18071154)
Supplement: Supplementary file 1 [file nutrients-18-01154-s001.zip › nutrients-4129038-supplementary.pdf]

Table S1. Fruit and vegetable intake by sociodemographic, behavioural, and regional subgroups: median (IQR) and mean (95% CI) daily servings.

Variable	Overall		Fruit intake	Vegetable intake	Combined fruit and vegetable intake	Fruit intake	Vegetable intake	Combined fruit and vegetable intake
	n	%	median (IQR), servings/d			mean (95% CI), servings/d		
Total	6720	100	1.0 (0.6-2.0)	1.1 (0.9-2.0)	2.3 (1.4-4.0)	1.3 (1.2-1.3)	1.7 (1.6-1.7)	3.0 (2.9-3.1)
**Gender**								
Men	3365	50.1	0.9 (0.4-1.7)	1.0 (0.7-2.0)	2.1 (1.4-3.7)	1.2 (1.2-1.3)	1.6 (1.5-1.6)	2.8 (2.7-2.9)
Women	3355	49.9	1.0 (0.6-2.0)	1.1 (0.9-2.0)	2.6 (1.6-4.0)	1.4 (1.4-1.5)	1.7 (1.7-1.8)	3.1 (3.0-3.2)
**Age groups**								
18-24	919	13.7	1.0 (0.6-2.0)	1.1 (0.9-2.0)	2.3 (1.4–3.9)	1.3 (1.3-1.4)	1.6 (1.5-1.7)	2.9 (2.8-3.0)
25-34	1641	24.4	1.0 (0.6-2.0)	1.3 (0.9-2.0)	2.4 (1.4–4.0)	1.4 (1.3-1.4)	1.7 (1.7-1.8)	3.1 (3.0-3.2)
35-44	1525	22.7	1.0 (0.4-2.0)	1.3 (0.9-2.0)	2.3 (1.4–4.0)	1.3 (1.2-1.3)	1.7 (1.6-1.7)	2.9 (2.8-3.0)
45-54	1268	18.9	1.0 (0.6-2.0)	1.0 (0.7-2.0)	2.3 (1.4–4.0)	1.3 (1.3-1.4)	1.6 (1.6-1.7)	2.9 (2.8-3.1)
55+	1367	20.3	1.0 (0.6-2.0)	1.0 (0.7-2.0)	2.3 (1.4–3.9)	1.4 (1.3-1.4)	1.6 (1.5-1.7)	2.9 (2.8-3.0)
**Ethnicity**								
Turkic	4730	70.4	1.0 (0.4-2.0)	1.1 (0.9-2.0)	2.3 (1.4-4.0)	1.4 (1.3-1.4)	1.7 (1.6-1.7)	3.0 (2.9-3.0)
Slavic	1660	24.7	1.0 (0.6-2.0)	1.1 (0.9-2.0)	2.3 (1.6-3.9)	1.2 (1.2-1.3)	1.6 (1.6-1.7)	2.8 (2.7-2.9)
Others	323	4.8	1.0 (0.6-2.0)	1.1 (0.9-1.8)	2.4 (1.6-4.0)	1.4 (1.2-1.5)	1.8 (1.6-1.9)	3.2 (2.9-3.4)
No answer	7	0.1	0.4 (0.4-1.2)	0.7 (0.5-1.8)	1.1 (0.9-3.0)	0.7 (0.1-1.3)	1.1 (0.1-2.1)	1.7 (0.1-3.4)
**Marital status**								
Married/Cohabiting	4519	67.2	1.0 (0.6-2.0)	1.1 (0.9-2.0)	2.3 (1.4-4.0)	1.4 (1.3-1.4)	1.7 (1.6-1.7)	3.0 (2.9-3.1)
Single	2192	32.6	1.0 (0.5-2.0)	1.1 (0.9-2.0)	2.3 (1.4-3.7)	1.3 (1.2-1.3)	1.6 (1.5-1.6)	2.9 (2.8-2.9)
No answer	9	0.1	0.6 (0.4-1.3)	1.4 (0.6-1.6)	2.0 (1.1-2.6)	0.9 (0.2-1.6)	1.4 (0.6-2.2)	2.3 (0.9-3.8)
**Education level**								
Primary education	83	1.2	1.0 (0.6-2.0)	1.3 (0.6-2.6)	2.1 (1.4-4.0)	1.3 (1.0-1.6)	1.7 (1.4-2.1)	3.0 (2.5-3.6)
Secondary education	2207	32.8	1.0 (0.4-2.0)	1.0 (0.7-2.0)	2.1 (1.4-4.0)	1.3 (1.3-1.4)	1.6 (1.5-1.7)	3.0 (2.9-3.0)

Higher education	4316	64.2	1.0 (0.6-2.0)	1.1 (0.9-2.0)	2.4 (1.6-4.0)	1.3 (1.3-1.4)	1.7 (1.7-1.7)	3.0 (2.9-3.0)
no answer	114	1.7	1.0 (0.6-1.7)	1.0 (1.0-2.0)	2.3 (1.6-3.7)	1.3 (1.1-1.6)	1.7 (1.4-2.0)	3.0 (2.6-3.5)
**Occupation**								
Employees with formal income	4307	64.1	1.0 (0.4–2.0)	1.1 (0.9–2.0)	2.3 (1.4–3.9)	1.3 (1.2–1.3)	1.6 (1.6-1.7)	2.9 (2.8-3.0)
Entrepreneur	552	8.2	1.0 (0.6–2.0)	1.7 (1.0–2.3)	2.6 (1.7–4.1)	1.5 (1.4–1.7)	1.9 (1.8-2.0)	3.4 (3.2-3.6)
Student	291	4.3	1.0 (0.4–2.0)	1.1 (0.7–2.0)	2.1 (1.4–3.5)	1.3 (1.1–1.4)	1.5 (1.3-1.6)	2.7 (2.5-2.9)
Unemployed	893	13.3	1.0 (0.5–2.0)	1.1 (0.9–2.1)	2.4 (1.4–4.1)	1.5 (1.4–1.6)	1.8 (1.7-1.9)	3.3 (3.1-3.4)
Pensioner	648	9.6	1.0 (0.6–2.0)	1.0 (0.7–2.0)	2.1 (1.4–3.4)	1.3 (1.2–1.4)	1.5 (1.4-1.6)	2.8 (2.6-2.9)
no answer	29	0.4	1.0 (0.4–1.4)	1.1 (0.7–2.0)	2.0 (1.5–3.5)	1.2 (0.6–1.8)	1.6 (1.1-2.1)	2.8 (1.7-3.9)
**Body Mass Index**								
Underweight (< 18.5 kg/m²)	201	3.0	1.0 (0.6-2.0)	2.0 (1.0–2.1)	2.7 (1.6–4.0)	1.5 (1.3-1.8)	1.9 (1.7–2.1)	3.4 (3.0–3.9)
Normal (18.5-24.99 kg/m²)	2593	38.6	1.0 (0.4-2.0)	1.1 (0.9–2.0)	2.3 (1.4–4.0)	1.3 (1.2-1.3)	1.6 (1.6–1.7)	2.9 (2.8–3.0)
Overweight (25.0-29.9 kg/m²)	2387	35.5	1.0 (0.6-2.0)	1.0 (0.7–2.0)	2.3 (1.4–4.0)	1.3 (1.3-1.4)	1.6 (1.6–1.7)	2.9 (2.8–3.0)
Obesity (≥30.0 kg/m²)	1374	20.4	1.0 (0.6-2.0)	1.1 (0.9–2.0)	2.4 (1.6–4.0)	1.3 (1.3-1.4)	1.7 (1.6–1.7)	3.0 (2.9–3.1)
no answer	165	2.5	1.0 (0.4-2.0)	1.4 (0.9–2.6)	2.9 (1.6–4.0)	1.4 (1.2-1.6)	1.8 (1.6–2.1)	3.3 (2.8–3.7)
**Smokers**								
Smokers	1284	19.1	0.9 (0.4–1.4)	1.1 (0.9–2.0)	2.1 (1.4–3.6)	1.1 (1.0–1.2)	1.6 (1.5–1.7)	2.7 (2.6–2.8)
Non-smokers	5436	80.9	1.0 (0.6–2.0)	1.1 (0.9–2.0)	2.4 (1.4–4.0)	1.4 (1.3–1.4)	1.7 (1.6–1.7)	3.0 (3.0–3.1)
**HED**								
No	6168	91.8	1.0 (0.57–2.0)	1.1 (0.9–2.0)	2.3 (1.4–4.0)	1.3 (1.3–1.4)	1.7 (1.6–1.7)	3.0 (2.9–3.0)
Yes	552	8.2	0.9 (0.43–2.0)	1.1 (0.9–2.0)	2.3 (1.4–4.0)	1.2 (1.1–1.3)	1.7 (1.5–1.8)	2.9 (2.7–3.0)
**Region**								
Astana city	448	6.7	0.9 (0.4–1.6)	1.0 (0.7–2.0)	2.1 (1.4–3.3)	1.2 (1.1–1.2)	1.4 (1.3–1.5)	2.3 (2.1–2.4)
Almaty city	560	8.3	0.9 (0.4–1.4)	1.0 (1.0–2.0)	2.1 (1.4–3.3)	1.1 (1.0–1.2)	1.5 (1.4–1.6)	3.2 (2.9–3.5)

Akmola	336	5.0	1.0 (0.4–1.7)	1.4 (1.0–2.0)	2.4 (1.6–3.9)	1.2 (1.1–1.3)	1.7 (1.6–1.8)	2.9 (2.6–3.2)
Aktobe	336	5.0	1.1 (0.9–1.7)	1.4 (0.9–3.0)	2.9 (2.0–4.5)	1.4 (1.3–1.5)	2.2 (1.9–2.4)	3.5 (3.3–3.7)
Almaty	560	8.3	0.9 (0.4–1.4)	1.0 (0.4–1.4)	1.9 (1.0–3.0)	1.2 (1.1–1.3)	1.2 (1.1–1.3)	3.0 (2.8–3.1)
Atyrau	336	5.0	1.3 (0.7–2.0)	1.7 (0.9–2.1)	3.0 (1.6–4.3)	1.5 (1.4–1.7)	1.8 (1.6–1.9)	2.4 (2.3–2.6)
West Kazakhstan	224	3.3	1.0 (0.6–2.0)	1.1 (0.6–2.0)	2.3 (1.4–4.0)	1.3 (1.1–1.4)	1.6 (1.4–1.8)	2.6 (2.4–2.8)
Zhambyl	448	6.7	1.0 (0.6–2.0)	2.0 (1.0–2.6)	3.0 (2.0–4.5)	1.5 (1.4–1.7)	2.0 (1.9–2.1)	3.4 (3.1–3.7)
Karaganda	448	6.7	1.0 (0.6–2.0)	1.4 (1.0–2.0)	2.6 (1.7–4.0)	1.3 (1.2–1.4)	1.6 (1.5–1.7)	3.3 (3.1–3.6)
Kostanay	336	5.0	0.9 (0.4–1.1)	1.0 (0.6–2.0)	2.1 (1.4–3.0)	1.0 (0.9–1.1)	1.4 (1.3–1.6)	2.7 (2.5–2.9)
Kyzylorda	336	5.0	0.9 (0.4–1.4)	1.4 (0.9–2.0)	2.3 (1.4–3.0)	1.1 (1.0–1.2)	1.5 (1.4–1.6)	2.4 (2.2–2.6)
Mangystau	336	5.0	1.1 (0.6–2.1)	1.4 (0.9–2.9)	2.9 (1.6–5.0)	1.6 (1.5–1.8)	1.8 (1.6–1.9)	2.7 (2.5–2.9)
Turkestan	560	8.3	1.0 (0.4–2.0)	1.1 (0.9–2.4)	2.6 (1.4–4.3)	1.5 (1.4–1.6)	1.9 (1.7–2.0)	4.4 (4.1–4.7)
Pavlodar	336	5.0	1.0 (0.6–1.5)	1.1 (0.9–2.0)	2.3 (1.7–3.4)	1.2 (1.1–1.3)	1.6 (1.4–1.7)	2.3 (2.1–2.4)
North Kazakhstan	224	3.3	1.0 (0.4–1.6)	1.0 (0.6–1.4)	2.0 (1.3–3.0)	1.1 (1.0–1.2)	1.2 (1.1–1.3)	3.2 (2.9–3.5)
East Kazakhstan	448	6.7	0.9 (0.4–1.3)	1.0 (0.6–2.0)	2.0 (1.3–3.1)	1.1 (1.0–1.3)	1.5 (1.4–1.6)	2.9 (2.6–3.2)
Shymkent city	448	6.7	1.4 (0.9–2.6)	2.0 (1.0–3.0)	3.4 (2.0–5.6)	2.1 (2.0–2.3)	2.3 (2.1–2.5)	3.5 (3.3–3.7)

IQR = interquartile range, CI = confidence interval, HED = heavy episodic drinking

Table S2. Minimally adjusted logistic regression models for adequate fruit and vegetable intake and salt-related knowledge, attitudes and behaviours by socio-demographic characteristics.

Exposure (independent variable)	Adjusted for (confounders only)	Category / Reference	aOR (95% CI)	p-value
**Outcome (dependent variable) - Adequate fruit & vegetable intake**				
Gender	Age group. Region	Men	0.72 (0.62–0.84)	0.001
		Women (Reference)	1.00	—
Age group	Gender. Region	18–24	0.93 (0.72–1.20)	0.569
		25–34	1.11 (0.89–1.38)	0.354
		35–44	0.90 (0.69–1.10)	0.238
		45–54	1.06 (0.84–1.33)	0.642
		55+ (Reference)	1.00	—
Ethnicity	Gender. Age group. Region	Turkic	1.14 (0.94–1.38)	0.191
		Slavic (Reference)	1.00	—
Marital status	Gender. Age group. Region	Married / Cohabiting	1.10 (0.93–1.32)	0.259
		Single (Reference)	1.00	—
Education level	Gender. Age group. Region	Primary education	0.90 (0.50–1.60)	0.730
		Secondary education	0.89 (0.75–1.05)	0.177
		Higher education (Reference)	1.00	—
Occupation	Gender. Age group. Region. Education level	Employees with formal income	1.15 (0.83–1.59)	0.391
		Entrepreneur	1.92 (1.30–2.83)	0.001
		Student	1.27 (0.74–2.19)	0.381
		Unemployed	1.52 (1.06–2.17)	0.021
		Pensioner (Reference)	1.00	—
Body Mass Index	Gender. Age group. Region.	Underweight (< 18.5 Kg/m²)	0.75 (0.51–1.10)	0.144
		Normal (18.5-24.99 Kg/m²)	0.81 (0.68–0.95)	0.010
		Overweight (25.0–29.9 kg/m²)	0.78 (0.66–0.91)	0.002
		Obesity (≥30 kg/m²) (Reference)	1.00	—
Smokers	Gender. Age group. Region. Education level	Smokers	0.78 (0.63–0.97)	0.028
		Non-smokers (Reference)	1.00	—
HED	Gender. Age group. Region. Education level	No	0.85 (0.68–1.07)	0.163
		Yes	1.00	—
Region	Gender. Age group. Education level	Astana city	0.19 (0.13–0.28)	0.001
		Almaty city	0.18 (0.12–0.26)	0.001
		Akmola	0.31 (0.21–0.46)	0.001
		Aktobe	0.55 (0.39–0.78)	0.001
		Almaty	0.28 (0.20–0.40)	0.001
		Atyrau	0.40 (0.26–0.62)	0.001
		West Kazakhstan	0.41 (0.27–0.61)	0.001
		Zhambyl	0.51 (0.37–0.71)	0.001

		Karaganda	0.28 (0.19–0.40)	0.001
		Kostanay	0.16 (0.10–0.26)	0.001
		Kyzylorda	0.24 (0.15–0.38)	0.001
		Mangystau	0.65 (0.46–0.92)	0.015
		Turkestan	0.63 (0.47–0.85)	0.002
		Pavlodar	0.20 (0.12–0.31)	0.001
		North Kazakhstan	0.20 (0.11–0.33)	0.001
		East Kazakhstan	0.26 (0.18–0.37)	0.001
		Shymkent city	1.00	—
**Outcome (dependent variable) - Salt-related knowledge (awareness of health risks of salt)**				
Gender	Age group. Region	Men	0.62 (0.54–0.71)	0.001
		Women (Reference)	1.00	—
Age group	Gender. Region	18–24	0.75 (0.60–0.96)	0.020
		25–34	0.79 (0.64–0.97)	0.022
		35–44	0.72 (0.58–0.88)	0.002
		45–54	1.05 (0.84–1.32)	0.647
		55+ (Reference)	1.00	—
Ethnicity	Gender. Age group. Region	Turkic	0.98 (0.83–1.16)	0.836
		Slavic (Reference)	1.00	—
Marital status	Gender. Age group. Region	Married / Cohabiting	0.94 (0.80–1.11)	0.475
		Single (Reference)	1.00	—
Education level	Gender. Age group. Region	Primary education	1.26 (0.60–2.61)	0.541
		Secondary education	0.77 (0.66–0.89)	0.001
		Higher education (Reference)	1.00	—
Occupation	Gender. Age group. Region. Education level	Employees with formal income	0.81 (0.60–1.10)	0.175
		Entrepreneur	0.97 (0.66–1.43)	0.891
		Student	0.71 (0.44–1.13)	0.149
		Unemployed	0.88 (0.62–1.24)	0.464
		Pensioner (Reference)	1.00	—
Body Mass Index	Gender. Age group. Region.	Underweight (< 18.5 Kg/m²)	0.77 (0.50–1.20)	0.255
		Normal (18.5-24.99 Kg/m²)	0.69 (0.57–0.85)	0.000
		Overweight (25.0–29.9 kg/m²)	0.74 (0.61–0.90)	0.002
		Obesity (≥30 kg/m²) (Reference)	1.00	—
HTN status	Gender. Age group. Region. Education level	Normotensive	0.66 (0.53–0.82)	0.001
		Pre-hypertensive	0.57 (0.46–0.70)	0.001
		Hypertensive	1.00	—
Smokers	Gender. Age group. Region. Education level	Smokers	1.00 (0.83–1.19)	0.976
		Non-smokers (Reference)	1.00	—
HED		No	1.03 (0.81–1.32)	0.794

	Gender. Age group. Region. Education level	Yes	1.00	—
Region	Gender. Age group. Education level	Astana city	0.55 (0.36–0.84)	0.006
		Almaty city	0.52 (0.35–0.78)	0.002
		Akmola	0.38 (0.24–0.59)	0.001
		Aktobe	0.16 (0.11–0.24)	0.001
		Almaty	0.26 (0.18–0.38)	0.001
		Atyrau	0.51 (0.31–0.85)	0.009
		West Kazakhstan	0.53 (0.31–0.89)	0.017
		Zhambyl	0.35 (0.23–0.53)	0.001
		Karaganda	0.47 (0.31–0.71)	0.001
		Kostanay	0.38 (0.25–0.59)	0.001
		Kyzylorda	0.25 (0.16–0.38)	0.001
		Mangystau	0.32 (0.21–0.49)	0.001
		Turkestan	0.52 (0.34–0.78)	0.002
		Pavlodar	0.53 (0.34–0.83)	0.005
		North Kazakhstan	0.39 (0.24–0.62)	0.001
		East Kazakhstan	0.82 (0.52–1.30)	0.402
		Shymkent city	1.00	—
**Outcome (dependent variable) - Salt-related attitude**				
Gender	Age group. Region	Men	0.74 (0.66–0.82)	0.001
		Women (Reference)	1.00	—
Age group	Gender. Region	18–24	0.38 (0.32–0.46)	0.001
		25–34	0.47 (0.40–0.55)	0.001
		35–44	0.61 (0.52–0.72)	0.001
		45–54	0.78 (0.66–0.93)	0.004
		55+ (Reference)	1.00	—
Ethnicity	Gender. Age group. Region	Turkic	1.04 (0.91–1.19)	0.572
		Slavic (Reference)	1.00	—
Marital status	Gender. Age group. Region	Married / Cohabiting	0.96 (0.84–1.09)	0.524
		Single (Reference)	1.00	—
Education level	Gender. Age group. Region	Primary education	0.78 (0.47–1.29)	0.335
		Secondary education	0.83 (0.74–0.95)	0.005
		Higher education (Reference)	1.00	—
Occupation	Gender. Age group. Region. Education level	Employees with formal income	0.73 (0.58–0.92)	0.007
		Entrepreneur	0.74 (0.55–0.99)	0.042
		Student	0.82 (0.55–1.21)	0.316
		Unemployed	0.74 (0.57–0.96)	0.026
		Pensioner (Reference)	1.00	—
Body Mass Index	Gender. Age group. Region.	Underweight (< 18.5 Kg/m²)	0.94 (0.66–1.32)	0.718
		Normal (18.5-24.99 Kg/m²)	0.97 (0.84–1.13)	0.702
		Overweight (25.0–29.9 kg/m²)	1.03 (0.89–1.19)	0.735

		Obesity (≥30 kg/m²) (Reference)	1.00	—
HTN status	Gender. Age group. Region. Education level	Normotensive	0.92 (0.78–1.08)	0.312
		Pre-hypertensive	0.91 (0.78–1.06)	0.231
		Hypertensive (Reference)	1.00	—
Smokers	Gender. Age group. Region. Education level	Smokers	0.65 (0.56–0.76)	0.001
		Non-smokers (Reference)	1.00	—
HED	Gender. Age group. Region. Education level	No	1.31 (1.07–1.61)	0.010
		Yes	1.00	—
Region	Gender. Age group. Education level	Astana city	0.59 (0.45–0.79)	0.001
		Almaty city	0.68 (0.52–0.89)	0.005
		Akmola	0.70 (0.51–0.97)	0.031
		Aktobe	1.81 (1.30–2.50)	0.001
		Almaty	0.72 (0.55–0.94)	0.016
		Atyrau	1.20 (0.84–1.71)	0.322
		West Kazakhstan	0.69 (0.49–0.99)	0.042
		Zhambyl	0.65 (0.48–0.87)	0.003
		Karaganda	0.72 (0.54–0.95)	0.019
		Kostanay	0.64 (0.47–0.88)	0.005
		Kyzylorda	1.01 (0.73–1.39)	0.977
		Mangystau	0.61 (0.44–0.85)	0.003
		Turkestan	1.05 (0.80–1.38)	0.714
		Pavlodar	0.52 (0.38–0.71)	0.001
		North Kazakhstan	0.68 (0.48–0.96)	0.030
		East Kazakhstan	0.65 (0.49–0.87)	0.004
		Shymkent city	1.00	—
**Outcome (dependent variable) - Salt-related behaviour**				
Gender	Age group. Region	Men	0.88 (0.78–0.98)	0.026
		Women (Reference)	1.00	—
Age group	Gender. Region	18–24	0.54 (0.44–0.66)	0.001
		25–34	0.61 (0.51–0.73)	0.001
		35–44	0.83 (0.70–0.98)	0.028
		45–54	0.86 (0.72–1.02)	0.086
		55+ (Reference)	1.00	—
Ethnicity	Gender. Age group. Region	Turkic	1.12 (0.97–1.29)	0.129
		Slavic (Reference)	1.00	—
Marital status	Gender. Age group. Region	Married / Cohabiting	0.97 (0.85–1.11)	0.678
		Single (Reference)	1.00	—
Education level	Gender. Age group. Region	Primary education	1.12 (0.66–1.89)	0.684
		Secondary education	0.87 (0.76–0.99)	0.038
		Higher education (Reference)	1.00	—
Occupation	Gender. Age group. Region. Education level	Employees with formal income	0.78 (0.61–0.98)	0.034
		Entrepreneur	0.60 (0.44–0.82)	0.001
		Student	0.98 (0.65–1.48)	0.915
		Unemployed	1.00 (0.76–1.31)	0.997

		Pensioner (Reference)	1.00	—
Body Mass Index	Gender. Age group. Region.	Underweight (< 18.5 Kg/m²)	1.32 (0.92–1.89)	0.132
		Normal (18.5-24.99 Kg/m²)	1.16 (0.99–1.35)	0.075
		Overweight (25.0–29.9 kg/m²)	1.03 (0.89–1.21)	0.679
		Obesity (≥30 kg/m²) (Reference)	1.00	—
HTN status	Gender. Age group. Region. Education level	Normotensive	1.02 (0.86–1.21)	0.844
		Pre-hypertensive	0.96 (0.81–1.13)	0.621
		Hypertensive (Reference)	1.00	—
Smokers	Gender. Age group. Region. Education level	Smokers	0.63 (0.53–0.74)	0.001
		Non-smokers (Reference)	1.00	—
HED	Gender. Age group. Region. Education level	No	1.43 (1.13–1.79)	0.002
		Yes	1.00	—
Region	Gender. Age group. Education level	Astana city	1.46 (1.10–1.95)	0.010
		Almaty city	0.95 (0.72–1.26)	0.732
		Akmola	1.21 (0.88–1.68)	0.244
		Aktobe	1.49 (1.08–2.05)	0.015
		Almaty	0.43 (0.32–0.59)	0.001
		Atyrau	0.60 (0.40–0.90)	0.013
		West Kazakhstan	1.31 (0.91–1.87)	0.142
		Zhambyl	0.81 (0.59–1.11)	0.191
		Karaganda	1.01 (0.75–1.36)	0.935
		Kostanay	0.78 (0.56–1.08)	0.137
		Kyzylorda	0.73 (0.52–1.04)	0.086
		Mangystau	1.03 (0.74–1.44)	0.867
		Turkestan	1.00 (0.75–1.34)	0.970
		Pavlodar	0.96 (0.69–1.33)	0.809
		North Kazakhstan	1.06 (0.73–1.52)	0.770
		East Kazakhstan	0.58 (0.42–0.79)	0.001
		Shymkent city	1.00	—

OR - odds ratio, CI = confidence interval, HED = heavy episodic drinking, HTN = hypertension

Table S3. Association between socio-demographic characteristics and knowledge, attitudes, and behaviours related to salt.

Variable	Knowledge		Attitude					Behaviour								
	High salt intake causes health problems		Self-assessment of salt intake			The importance of reducing salt intake		Salt addition during eating			Addition of salt in food preparation			Consumption of salty processed foods		
	Yes	No / Don't know	Very high / High	Normal	Very low / low	Very important	Not important at all / Completely unimportant	Always/Often	Sometimes	Rarely /Never	Always/Often	Sometimes	Rarely /Never	Always/Often	Sometimes	Rarely/ Never
**Total**	80.6 (79.6-81.5)	19.4 (18.5-20.4)	13.7 (12.9–14.5)	64.4 (63.3–65.6)	21.9 (20.9–22.9)	38.3(37.2–39.5)	61.7(60.5–62.8)	39.9 (38.8–41.1)	20.0 (19.0–20.9)	40.1 (38.9–41.3)	63.2 (62.0–64.3)	20.4 (19.5–21.4)	16.4 (15.5–17.3)	30.2 (29.1–31.3)	34.0 (32.9–35.2)	35.8 (34.6–36.9)
**Gender**																
Men	78.0 (76.6–79.4)	22.0 (20.6–23.4)	13.8(12.7–15.0)	66.1(64.5–67.7)	20.1(18.7–21.4)	33.8 (32.2–35.4)	66.2 (64.6–67.8)	38.7 (37.0–40.3)	21.4 (20.0–22.8)	39.9 (38.3–41.6)	63.8 (62.1–65.4)	21.2 (19.8–22.6)	15.0 (13.9–16.3)	32.2 (30.7–33.8)	34.4 (32.9–36.1)	33.3 (31.8–34.9)
Women	83.2 (81.9–84.5)	16.8 (15.5–18.1)	13.6(12.4–14.8)	62.7(61.1–64.3)	23.7(22.3–25.2)	43.0 (41.3–44.6)	57.0 (55.4–58.7)	41.2 (39.6–42.9)	18.5 (17.2–19.9)	40.3 (38.6–41.9)	62.6 (60.9–64.2)	19.7 (18.4–21.0)	17.7 (16.5–19.1)	28.2 (26.7–29.7)	33.6 (32.0–35.2)	38.2 (36.6–39.9)
χ2 (p)	29.5 (<0.001)		13.4 (0.001)			60.0 (0.001)		9.8 (0.007)			9.7 (0.008)			20.7 (<0.001)		
**Age groups**																
18-24	78.5 (75.8–81.1)	21.5 (18.9–24.2)	16.8 (14.4–19.3)	66.9 (63.8–69.9)	16.3 (14.0–18.8)	30.9 (28.0–33.9)	69.1 (66.1–72.0)	41.6 (38.4–44.8)	22.5 (19.9–25.3)	35.9 (32.9–39.1)	67.2 (64.2–70.2)	19.8 (17.3–22.5)	12.9 (10.9–15.2)	35.3 (32.2–38.4)	36.0 (33.0–39.2)	28.7 (25.9–31.7)
25-34	80.7 (78.8–82.6)	19.3 (17.4–21.2)	16.3 (14.6–18.2)	64.6 (62.3–66.9)	19.1 (17.2–21.0)	33.4 (31.1–35.7)	66.6 (64.3–68.9)	42.7 (40.3–45.1)	20.2 (18.3–22.2)	37.1 (34.8–39.5)	66.8 (64.5–69.0)	20.2 (18.3–22.2)	13.0 (11.4–14.7)	33.8 (31.6–36.1)	34.5 (32.2–36.8)	31.7 (29.5–34.0)

35-44	77.8 (75.7–79.9)	22.2 (20.1–24.3)	13.8 (12.1–15.6)	65.5 (63.1–67.9)	20.7 (18.7–22.8)	36.4 (34.0–38.8)	63.6 (61.2–66.0)	39.7 (37.3–42.2)	20.3 (18.4–22.4)	39.9 (37.5–42.4)	63.5 (61.0–65.9)	19.8 (17.9–21.9)	16.7 (14.9–18.7)	29.4 (27.2–31.8)	35.3 (32.9–37.7)	35.3 (32.9–37.7)
45-54	83.2 (81.1–85.3)	16.8 (14.7–18.9)	12.6 (10.9–14.5)	64.3 (61.6–66.9)	23.1 (20.9–25.5)	44.0 (41.3–46.7)	56.0 (53.3–58.7)	37.7 (35.1–40.4)	19.6 (17.4–21.8)	42.7 (40.0–45.5)	61.0 (58.3–63.6)	21.1 (18.9–23.4)	18.0 (15.9–20.2)	27.1 (24.7–29.6)	34.1 (31.6–36.8)	38.7 (36.1–41.4)
55+	82.7 (80.7–84.7)	17.3 (15.3–19.3)	9.4 (7.9–11.0)	61.4 (58.8–64.0)	29.2 (26.8–31.6)	46.2 (43.6–48.9)	53.8 (51.1–56.4)	37.8 (35.3–40.4)	17.9 (16.0–20.0)	44.3 (41.6–46.9)	57.9 (55.2–60.5)	21.2 (19.1–23.4)	20.9 (18.8–23.1)	26.1 (23.8–28.5)	30.6 (28.2–33.1)	43.3 (40.7–45.9)
χ2 (p)	19.7 (0.001)		91.5 (<0.001)			94.2 (<0.001)		28.8 (<0.001)			51.6 (<0.001)			78.6 (<0.001)		
**Ethnicity**																
Turkic	80.3 (79.1–81.4)	19.7 (18.6–20.9)	13.3 (12.4–14.3)	65.0 (63.7–66.4)	21.7 (20.5–22.9)	40.4 (39.0–41.8)	59.6 (58.2–61.0)	40.6 (39.2–42.0)	20.8 (19.7–22.0)	38.5 (37.2–39.9)	59.4 (58.0–60.8)	23.1 (21.9–24.3)	17.5 (16.4–18.6)	29.2 (28.0–30.5)	35.5 (34.2–36.9)	35.2 (33.9–36.6)
Slavic	81.6 (79.7–83.4)	18.4 (16.6–20.3)	14.5 (12.8–16.2)	62.4 (60.1–64.7)	23.1 (21.2–25.2)	34.2 (32.0–36.5)	65.8 (63.5–68.0)	37.8 (35.5–40.2)	17.9 (16.1–19.8)	44.3 (41.9–46.7)	72.3 (70.2–74.5)	13.9 (12.3–15.6)	13.7 (12.1–15.5)	32.4 (30.2–34.7)	30.1 (27.9–32.4)	37.5 (35.2–39.8)
Others	79.9 (75.2–84.0)	20.1 (16.0–24.8)	15.5 (11.9–19.7)	65.6 (60.3–70.7)	18.9 (14.9–23.4)	29.7 (24.9–34.9)	70.3 (65.1–75.1)	41.2 (35.9–46.6)	17.6 (13.8–22.1)	41.2 (35.9–46.6)	71.8 (66.7–76.5)	14.6 (11.0–18.7)	13.6 (10.2–17.7)	32.8 (27.9–38.1)	32.5 (27.6–37.8)	34.7 (29.6–40.0)
χ2 (p)	3.3 (0.351)		6.2 (0.397)			30.5 (<0.001)		20.8 (0.002)			106.5 (<0.001)			19.1 (0.004)		
**Marital status**																
Married /Cohabiting	80.2 (79.0–81.4)	19.8 (18.6–21.0)	13.3 (12.4–14.4)	65.1 (63.7–66.4)	21.6 (20.4–22.8)	39.7 (38.3–41.1)	60.3 (58.9–61.7)	40.1 (38.6–41.5)	19.8 (18.7–21.0)	40.1 (38.7–41.5)	61.4 (60.0–62.8)	21.7 (20.5–22.9)	16.9 (15.8–18.0)	29.7 (28.3–31.0)	34.4 (33.0–35.8)	36.0 (34.6–37.4)
Single	81.3 (79.6–82.9)	18.7 (17.1–20.4)	14.5 (13.0–16.0)	63.0 (60.9–65.0)	22.6 (20.9–24.4)	35.7 (33.7–37.7)	64.3 (62.3–66.3)	39.6 (37.6–41.7)	20.2 (18.5–21.9)	40.2 (38.2–42.3)	66.8 (64.8–68.8)	17.8 (16.2–19.4)	15.4 (13.9–16.9)	31.4 (29.5–33.4)	33.2 (31.3–35.2)	35.4 (33.4–37.4)
χ2 (p)	3.3 (0.195)		8.0 (0.092)			12.6 (0.002)		3.7 (0.451)			22.2 (<0.001)			4.5 (0.340)		
**Education level**																

Primary education	88.0 (79.7–93.6)	12.0 (6.4–20.3)	24.1 (15.9–34.1)	55.4 (44.7–65.8)	20.5 (12.9–30.1)	54.2 (43.5–64.6)	45.8 (35.4–56.5)	54.2 (43.5–64.6)	13.3 (7.3–21.8)	32.5 (23.2–43.1)	53.0 (42.3–63.5)	21.7 (13.9–31.4)	25.3 (16.9–35.4)	34.9 (25.3–45.6)	27.7 (19.0–38.0)	37.3 (27.5–48.0)
Secondary education	77.7 (75.9–79.4)	22.3 (20.6–24.1)	14.3 (12.9–15.8)	64.4 (62.4–66.4)	21.3 (19.6–23.0)	35.4 (33.5–37.4)	64.6 (62.6–66.5)	41.9 (39.9–44.0)	19.6 (18.0–21.3)	38.5 (36.5–40.5)	65.2 (63.1–67.1)	20.0 (18.4–21.7)	14.8 (13.4–16.3)	33.0 (31.1–35.0)	33.9 (31.9–35.9)	33.1 (31.1–35.1)
Higher education	81.9 (80.7–83.0)	18.1 (17.0–19.3)	13.2 (12.2–14.2)	64.9 (63.5–66.4)	21.9 (20.7–23.1)	39.4 (37.9–40.9)	60.6 (59.1–62.1)	38.7 (37.2–40.2)	20.2 (19.0–21.4)	41.1 (39.6–42.6)	62.6 (61.2–64.1)	20.6 (19.4–21.8)	16.8 (15.7–18.0)	28.7 (27.4–30.1)	34.5 (33.1–35.9)	36.8 (35.3–38.2)
No answer	82.5 (74.7–88.6)	17.5 (11.4–25.3)	14.0 (8.6–21.3)	50.9 (41.8–59.9)	35.1 (26.8–44.1)	43.9 (35.0–53.0)	56.1 (47.0–65.0)	38.6 (30.0–47.7)	21.1 (14.4–29.2)	40.4 (31.7–49.5)	53.5 (44.4–62.5)	22.8 (15.8–31.1)	23.7 (16.6–32.1)	27.2 (19.7–35.9)	22.8 (15.8–31.1)	50.0 (40.9–59.1)
χ2 (p)	19.5 (<0.001)		22.1 (0.001)			20.2 (<0.001)		14.0 (0.029)			16.8 (0.010)			27.6 (<0.001)		
**Occupation**																
Employees with formal income	80.1 (78.9–81.3)	19.9 (18.7–21.1)	14.0 (13.0–15.1)	65.0 (63.6–66.4)	21.0 (19.8–22.2)	37.4 (35.9–38.8)	62.6 (61.2–64.1)	39.3 (37.9–40.8)	20.5 (19.4–21.8)	40.1 (38.7–41.6)	63.6 (62.2–65.0)	20.8 (19.6–22.0)	15.6 (14.5–16.7)	30.7 (29.3–32.1)	34.9 (33.5–36.3)	34.5 (33.0–35.9)
Entrepreneur	80.8 (77.4–83.9)	19.2 (16.1–22.6)	15.2 (12.4–18.4)	63.9 (59.9–67.9)	20.8 (17.6–24.4)	34.4 (30.5–38.5)	65.6 (61.5–69.5)	42.4 (38.3–46.5)	19.4 (16.3–22.8)	38.2 (34.2–42.3)	67.9 (64.0–71.7)	17.6 (14.6–20.9)	14.5 (11.7–17.6)	33.5 (29.7–37.5)	32.8 (29.0–36.8)	33.7 (29.8–37.7)
Student	75.3 (70.1–79.9)	24.7 (20.1–29.9)	16.8 (12.9–21.5)	67.7 (62.2–72.9)	15.5 (11.7–20.0)	32.0 (26.8–37.5)	68.0 (62.5–73.2)	33.7 (28.4–39.2)	23.7 (19.1–28.8)	42.6 (37.0–48.3)	71.1 (65.7–76.1)	15.1 (11.4–19.6)	13.7 (10.2–18.1)	33.7 (28.4–39.2)	36.1 (30.7–41.7)	30.2 (25.2–35.7)
Unemployed	82.0 (79.3–84.4)	18.0 (15.6–20.7)	14.9 (12.7–17.3)	63.4 (60.2–66.5)	21.7 (19.1–24.5)	40.0 (36.8–43.2)	60.0 (56.8–63.2)	45.4 (42.1–48.6)	16.8 (14.5–19.4)	37.8 (34.7–41.1)	61.7 (58.5–64.8)	20.8 (18.3–23.6)	17.5 (15.1–20.1)	30.2 (27.3–33.3)	32.7 (29.7–35.8)	37.1 (33.9–40.3)
Pensioner	84.1 (81.1–86.8)	15.9 (13.2–18.9)	7.3 (5.4–9.4)	61.0 (57.2–64.7)	31.8 (28.3–35.5)	48.9 (45.1–52.8)	51.1 (47.2–54.9)	38.3 (34.6–42.1)	18.2 (15.4–21.3)	43.5 (39.7–47.4)	56.2 (52.3–60.0)	21.1 (18.1–24.4)	22.7 (19.6–26.0)	23.1 (20.0–26.5)	29.8 (26.4–33.4)	47.1 (43.2–50.9)

No answer	82.8 (66.3–93.1)	17.2 (6.9–33.7)	6.9 (1.5–20.3)	65.5 (47.4–80.7)	27.6 (14.0–45.4)	37.9 (22.1–56.0)	62.1 (44.0–77.9)	20.7 (9.1–37.8)	41.4 (25.0–59.4)	37.9 (22.1–56.0)	31.0 (16.6–49.0)	44.8 (27.9–62.7)	24.1 (11.5–41.6)	17.2 (6.9–33.7)	44.8 (27.9–62.7)	37.9 (22.1–56.0)
χ2 (p)	12.3 (0.031)		64.0 (<0.001)			42.1 (<0.001)		32.8 (<0.001)			51.9 (<0.001)			51.0 (<0.001)		
**Body Mass Index**																
Under weight (< 18.5 kg/m²)	86.6 (81.3–90.7)	13.4 (9.3–18.7)	14.4 (10.1–19.8)	63.7 (56.9–70.1)	21.9 (16.6–28.0)	34.3 (28.0–41.1)	65.7 (58.9–72.0)	37.3 (30.8–44.1)	17.9 (13.1–23.6)	44.8 (38.0–51.7)	68.2 (61.5–74.3)	13.9 (9.7–19.2)	17.9 (13.1–23.6)	29.4 (23.4–35.9)	33.8 (27.6–40.6)	36.8 (30.4–43.6)
Normal (18.5-24.99 kg/m²)	83.0 (81.5–84.4)	17.0 (15.6–18.5)	14.4 (13.1–15.8)	64.5 (62.7–66.3)	21.1 (19.6–22.7)	38.7 (36.8–40.6)	61.3 (59.4–63.2)	40.5 (38.6–42.4)	19.3 (17.8–20.9)	40.2 (38.3–42.1)	66.7 (64.9–68.5)	19.1 (17.6–20.7)	14.2 (12.9–15.5)	31.0 (29.2–32.8)	32.6 (30.8–34.4)	36.4 (34.6–38.3)
Overweight (25.0-29.9 kg/m²)	81.3 (79.7–82.8)	18.7 (17.2–20.3)	13.4 (12.1–14.8)	63.8 (61.8–65.7)	22.8 (21.2–24.5)	38.5 (36.5–40.4)	61.5 (59.6–63.5)	41.9 (40.0–43.9)	18.0 (16.5–19.6)	40.1 (38.1–42.0)	63.0 (61.0–64.9)	20.2 (18.6–21.8)	16.9 (15.4–18.4)	29.6 (27.8–31.5)	34.7 (32.8–36.6)	35.7 (33.8–37.6)
Obesity (≥30.0 kg/m²)	74.2 (71.8–76.4)	25.8 (23.6–28.2)	12.4 (10.8–14.3)	65.4 (62.8–67.8)	22.2 (20.1–24.5)	39.2 (36.6–41.8)	60.8 (58.2–63.4)	36.3 (33.8–38.9)	24.2 (22.0–26.5)	39.5 (37.0–42.1)	56.6 (54.0–59.2)	23.4 (21.3–25.7)	19.9 (17.9–22.1)	29.3 (27.0–31.8)	35.2 (32.7–37.7)	35.5 (33.0–38.1)
No answer	79.4 (72.7–85.0)	20.6 (15.0–27.3)	16.4 (11.3–22.6)	65.5 (58.0–72.4)	18.2 (12.9–24.6)	29.7 (23.1–37.0)	70.3 (63.0–76.9)	35.8 (28.7–43.3)	25.5 (19.3–32.5)	38.8 (31.6–46.4)	59.4 (51.8–66.7)	27.9 (21.5–35.1)	12.7 (8.3–18.4)	33.9 (27.0–41.4)	37.6 (30.5–45.1)	28.5 (22.0–35.7)
χ2 (p)	51.3 (<0.001)		6.8 (0.563)			7.1 (0.130)		29.6 (<0.001)			53.1 (<0.001)			7.8 (0.451)		
**HTN status**																
Normotensive	82.6 (81.0–84.2)	17.4 (15.8–19.0)	14.1 (12.7–15.6)	64.0 (62.0–66.0)	21.9 (20.2–23.6)	35.0 (33.0–37.0)	65.0 (63.0–67.0)	41.2 (39.1–43.2)	18.9 (17.3–20.5)	40.0 (37.9–42.0)	64.7 (62.7–66.7)	19.3 (17.7–21.0)	15.9 (14.5–17.5)	30.4 (28.5–32.4)	33.2 (31.3–35.2)	36.3 (34.4–38.4)
Pre-hypertensive	77.7 (76.0–79.3)	22.3 (20.7–24.0)	13.1 (11.8–14.5)	67.0 (65.1–68.9)	19.9 (18.3–21.5)	35.4 (33.5–37.4)	64.6 (62.6–66.5)	40.1 (38.1–42.1)	21.2 (19.6–22.9)	38.7 (36.8–40.7)	63.5 (61.6–65.5)	21.2 (19.6–22.9)	15.3 (13.8–16.8)	28.9 (27.1–30.7)	37.9 (36.0–39.9)	33.2 (31.3–35.1)

Hypertensive	81.9 (80.2–83.5)	18.1 (16.5–19.8)	13.9 (12.5–15.5)	61.8 (59.7–63.9)	24.2 (22.4–26.1)	45.4 (43.3–47.6)	54.6 (52.4–56.7)	38.6 (36.5–40.7)	19.8 (18.1–21.5)	41.7 (39.6–43.8)	61.1 (59.0–63.2)	20.7 (19.0–22.4)	18.2 (16.6–19.9)	31.4 (29.4–33.4)	30.4 (28.5–32.4)	38.2 (36.1–40.3)
χ2 (p)	20.9 (<0.001)		15.4 (0.004)			63.7 (<0.001)		7.3 (0.121)			10.6 (0.031)			29.3 (<0.001)		
**Smokers**																
Smokers	80.0 (77.7–82.1)	20.0 (17.9–22.3)	18.1 (16.1–20.3)	63.6 (60.9–66.2)	18.3 (16.3–20.5)	27.9 (25.5–30.4)	72.1 (69.6–74.5)	42.7 (40.0–45.4)	18.9 (16.9–21.1)	38.4 (35.8–41.1)	73.3 (70.8–75.7)	14.6 (12.7–16.6)	12.1 (10.4–14.0)	40.6 (37.9–43.3)	31.3 (28.8–33.9)	28.1 (25.7–30.6)
Non-smokers	80.7 (79.7–81.8)	19.3 (18.2–20.3)	12.6 (11.8–13.5)	64.6 (63.3–65.9)	22.7 (21.6–23.9)	40.8 (39.5–42.1)	59.2 (57.9–60.5)	39.3 (38.0–40.6)	20.2 (19.1–21.3)	40.5 (39.2–41.8)	60.8 (59.5–62.1)	21.8 (20.7–22.9)	17.4 (16.4–18.4)	27.7 (26.6–28.9)	34.7 (33.4–35.9)	37.6 (36.3–38.9)
χ2 (p)	0.4 (0.538)		32.5 (<0.001)			73.5 (<0.001)		5.0 (0.083)			69.8 (<0.001)			86.2 (<0.001)		
**HED**																
No	80.7 (79.7–81.7)	19.3 (18.3–20.3)	13.4 (12.5–14.2)	64.5 (63.3–65.7)	22.2 (21.1–23.2)	39.0 (37.8–40.2)	61.0 (59.8–62.2)	39.5 (38.3–40.7)	20.1 (19.1–21.1)	40.4 (39.2–41.6)	62.4 (61.2–63.6)	20.9 (19.9–21.9)	16.7 (15.8–17.6)	29.3 (28.1–30.4)	34.2 (33.0–35.4)	36.5 (35.3–37.8)
Yes	79.3 (75.8–82.6)	20.7 (17.4–24.2)	17.4 (14.4–20.7)	63.8 (59.7–67.7)	18.8 (15.7–22.3)	30.8 (27.1–34.7)	69.2 (65.3–72.9)	44.7 (40.6–48.9)	18.5 (15.4–21.9)	36.8 (32.8–40.9)	71.6 (67.7–75.2)	15.4 (12.6–18.6)	13.0 (10.4–16.0)	40.6 (36.5–44.7)	32.1 (28.3–36.0)	27.4 (23.8–31.2)
χ2 (p)	0.6 (0.439)		8.6 (0.013)			14.5 (<0.001)		5.8 (0.055)			18.2 (<0.001)			34.1 (<0.001)		
**Region**																
Astana city	84.4 (80.8–87.5)	15.6 (12.5–19.2)	12.3 (9.5–15.6)	64.7 (60.2–69.1)	23.0 (19.3–27.1)	28.3 (24.3–32.7)	71.7 (67.3–75.7)	29.9 (25.8–34.3)	13.4 (10.5–16.8)	56.7 (52.1–61.2)	61.6 (57.0–66.0)	14.1 (11.1–17.5)	24.3 (20.5–28.5)	28.3 (24.3–32.7)	31.5 (27.3–35.9)	40.2 (35.7–44.8)
Almaty city	83.4 (80.1–86.3)	16.6 (13.7–19.9)	18.2 (15.2–21.6)	54.8 (50.7–58.9)	27.0 (23.4–30.8)	32.0 (28.2–35.9)	68.0 (64.1–71.8)	28.2 (24.6–32.0)	13.4 (10.8–16.4)	58.4 (54.3–62.4)	90.5 (87.9–92.8)	5.0 (3.4–7.0)	4.5 (3.0–6.4)	34.1 (30.3–38.1)	24.5 (21.0–28.2)	41.4 (37.4–45.5)
Akmola	82.4 (78.1–86.2)	17.6 (13.8–21.9)	12.2 (9.0–16.0)	59.2 (53.9–64.4)	28.6 (23.9–33.6)	32.7 (27.9–37.9)	67.3 (62.1–72.1)	30.7 (25.9–35.7)	13.7 (10.3–17.7)	55.7 (50.3–60.9)	76.5 (71.7–80.8)	11.0 (8.0–14.7)	12.5 (9.3–16.4)	25.3 (20.9–30.1)	29.2 (24.5–34.2)	45.5 (40.3–50.9)

Aktobe	61.9 (56.6–67.0)	38.1 (33.0–43.4)	9.8 (7.0–13.3)	66.7 (61.5–71.5)	23.5 (19.2–28.3)	52.4 (47.0–57.7)	47.6 (42.3–53.0)	20.5 (16.5–25.1)	29.5 (24.8–34.5)	50.0 (44.7–55.3)	40.8 (35.6–46.1)	28.3 (23.7–33.3)	31.0 (26.2–36.0)	16.7 (13.0–20.9)	43.5 (38.2–48.8)	39.9 (34.8–45.2)
Almaty	71.4 (67.6–75.1)	28.6 (24.9–32.4)	10.4 (8.0–13.1)	72.0 (68.1–75.6)	17.7 (14.7–21.0)	42.9 (38.8–47.0)	57.1 (53.0–61.2)	58.4 (54.3–62.4)	25.4 (21.9–29.1)	16.3 (13.4–19.5)	53.6 (49.4–57.7)	31.8 (28.0–35.7)	14.6 (11.9–17.8)	27.1 (23.6–30.9)	45.5 (41.4–49.7)	27.3 (23.8–31.1)
Atyrau	74.1 (69.2–78.6)	25.9 (21.4–30.8)	13.1 (9.8–17.0)	63.7 (58.4–68.7)	23.2 (18.9–27.9)	43.8 (38.5–49.1)	56.3 (50.9–61.5)	59.2 (53.9–64.4)	24.4 (20.0–29.2)	16.4 (12.7–20.6)	46.4 (41.1–51.8)	33.6 (28.7–38.8)	19.9 (15.9–24.5)	36.6 (31.6–41.9)	36.0 (31.0–41.2)	27.4 (22.8–32.3)
West Kazakhstan	85.7 (80.7–89.8)	14.3 (10.2–19.3)	11.2 (7.5–15.8)	61.6 (55.1–67.8)	27.2 (21.7–33.3)	35.7 (29.7–42.1)	64.3 (57.9–70.3)	35.7 (29.7–42.1)	28.6 (23.0–34.7)	35.7 (29.7–42.1)	47.3 (40.9–53.9)	23.7 (18.5–29.5)	29.0 (23.4–35.2)	25.4 (20.1–31.4)	38.4 (32.2–44.9)	36.2 (30.1–42.6)
Zhambyl	80.6 (76.7–84.0)	19.4 (16.0–23.3)	10.9 (8.3–14.1)	70.3 (66.0–74.4)	18.8 (15.3–22.6)	32.6 (28.4–37.0)	67.4 (63.0–71.6)	31.5 (27.3–35.9)	21.0 (17.4–24.9)	47.5 (42.9–52.2)	73.2 (69.0–77.2)	18.3 (14.9–22.1)	8.5 (6.2–11.3)	36.8 (32.5–41.4)	32.8 (28.6–37.3)	30.4 (26.2–34.7)
Karaganda	81.0 (77.2–84.5)	19.0 (15.5–22.8)	16.5 (13.3–20.2)	58.7 (54.1–63.2)	24.8 (21.0–28.9)	35.0 (30.7–39.5)	65.0 (60.5–69.3)	37.9 (33.5–42.5)	17.6 (14.3–21.4)	44.4 (39.9–49.0)	75.9 (71.8–79.7)	11.6 (8.9–14.8)	12.5 (9.7–15.8)	27.5 (23.5–31.7)	30.1 (26.0–34.5)	42.4 (37.9–47.0)
Kostanay	80.1 (75.5–84.1)	19.9 (15.9–24.5)	14.9 (11.4–19.0)	61.0 (55.7–66.1)	24.1 (19.8–28.9)	34.2 (29.3–39.4)	65.8 (60.6–70.7)	37.8 (32.7–43.1)	13.7 (10.3–17.7)	48.5 (43.2–53.8)	78.9 (74.3–83.0)	9.5 (6.7–13.0)	11.6 (8.5–15.4)	28.0 (23.4–32.9)	31.5 (26.8–36.7)	40.5 (35.3–45.8)
Kyzylorda	77.7 (73.0–81.9)	22.3 (18.1–27.0)	18.8 (14.9–23.2)	65.5 (60.3–70.4)	15.8 (12.2–20.0)	49.7 (44.4–55.0)	50.3 (45.0–55.6)	46.1 (40.9–51.5)	27.7 (23.1–32.6)	26.2 (21.7–31.1)	45.8 (40.6–51.2)	34.5 (29.6–39.7)	19.6 (15.7–24.1)	42.6 (37.4–47.9)	27.7 (23.1–32.6)	29.8 (25.1–34.8)
Mangystau	75.3 (70.5–79.7)	24.7 (20.3–29.5)	12.5 (9.3–16.4)	66.1 (60.9–71.0)	21.4 (17.3–26.0)	28.0 (23.4–32.9)	72.0 (67.1–76.6)	24.1 (19.8–28.9)	30.1 (25.3–35.1)	45.8 (40.6–51.2)	67.9 (62.7–72.7)	27.1 (22.5–32.0)	5.1 (3.1–7.8)	28.3 (23.7–33.3)	39.0 (33.9–44.3)	32.7 (27.9–37.9)
Turkestan	85.9 (82.8–88.6)	14.1 (11.4–17.2)	13.9 (11.2–17.0)	63.2 (59.2–67.1)	22.9 (19.5–26.5)	46.1 (42.0–50.2)	53.9 (49.8–58.0)	51.2 (47.1–55.4)	25.2 (21.7–28.9)	23.6 (20.2–27.2)	40.7 (36.7–44.8)	34.1 (30.3–38.1)	25.2 (21.7–28.9)	24.3 (20.9–28.0)	43.2 (39.2–47.3)	32.5 (28.7–36.5)

Pavlodar	83.9 (79.7–87.6)	16.1 (12.4–20.3)	10.7 (7.7–14.4)	67.6 (62.4–72.4)	21.7 (17.6–26.4)	20.2 (16.2–24.8)	79.8 (75.2–83.8)	37.2 (32.2–42.5)	16.1 (12.4–20.3)	46.7 (41.4–52.1)	63.7 (58.4–68.7)	15.5 (11.9–19.6)	20.8 (16.8–25.4)	31.5 (26.8–36.7)	34.5 (29.6–39.7)	33.9 (29.0–39.1)
North Kazakhstan	79.0 (73.3–84.0)	21.0 (16.0–26.7)	16.1 (11.7–21.3)	60.7 (54.2–66.9)	23.2 (18.1–29.1)	33.9 (28.0–40.3)	66.1 (59.7–72.0)	39.7 (33.5–46.2)	12.9 (9.0–17.8)	47.3 (40.9–53.9)	80.8 (75.3–85.5)	10.3 (6.8–14.8)	8.9 (5.7–13.2)	31.7 (25.9–38.0)	23.2 (18.1–29.1)	45.1 (38.7–51.6)
East Kazakhstan	88.4 (85.2–91.1)	11.6 (8.9–14.8)	11.2 (8.5–14.3)	72.8 (68.5–76.7)	16.1 (12.9–19.7)	38.4 (34.0–43.0)	61.6 (57.0–66.0)	43.1 (38.6–47.7)	15.2 (12.1–18.7)	41.7 (37.2–46.3)	78.1 (74.1–81.8)	12.9 (10.1–16.3)	8.9 (6.6–11.8)	34.2 (29.9–38.6)	31.5 (27.3–35.9)	34.4 (30.1–38.9)
Shymkent city	89.7 (86.7–92.3)	10.3 (7.7–13.3)	18.8 (15.3–22.6)	63.8 (59.3–68.2)	17.4 (14.1–21.1)	59.2 (54.6–63.6)	40.8 (36.4–45.4)	54.9 (50.3–59.5)	15.2 (12.1–18.7)	29.9 (25.8–34.3)	48.9 (44.3–53.5)	24.3 (20.5–28.5)	26.8 (22.8–31.0)	33.9 (29.7–38.4)	31.0 (26.9–35.4)	35.0 (30.7–39.5)
χ2 (p)	187.7 (<0.001)		114.5 (<0.001)			259.7 (<0.001)		675.7 (< 0.001)			868.5 (< 0.001)			213.6 (< 0.001)		

CI = confidence interval, HED = heavy episodic drinking, HTN = hypertension
